# A Simple Iron-Catalyst for Alkenylation of Ketones Using Primary Alcohols

**DOI:** 10.3390/molecules25071590

**Published:** 2020-03-30

**Authors:** Motahar Sk, Ashish Kumar, Jagadish Das, Debasis Banerjee

**Affiliations:** Department of Chemistry, Laboratory of Catalysis and Organic Synthesis, Indian Institute of Technology Roorkee, Roorkee-247667, India; msk@cy.iitr.ac.in (M.S.); akumar5@cy.iitr.ac.in (A.K.); dasjagadish82@gmail.com (J.D.)

**Keywords:** α-alkenylation, iron, alcohols, dehydrogenative coupling, sustainability

## Abstract

Herein, we developed a simple iron-catalyzed system for the α-alkenylation of ketones using primary alcohols. Such acceptor-less dehydrogenative coupling (ADC) of alcohols resulted in the synthesis of a series of important α,β-unsaturated functionalized ketones, having aryl, heteroaryl, alkyl, nitro, nitrile and trifluoro-methyl, as well as halogen moieties, with excellent yields and selectivity. Initial mechanistic studies, including deuterium labeling experiments, determination of rate and order of the reaction, and quantitative determination of H_2_ gas, were performed. The overall transformations produce water and dihydrogen as byproducts.

## 1. Introduction

**Scheme 1:**
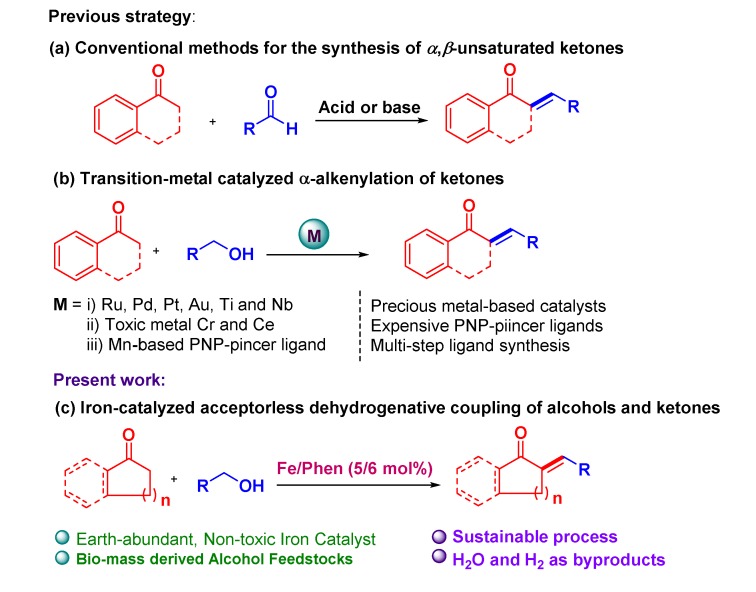
Approaches for the synthesis of α,β-unsaturated ketones. (**a**) Conventional methods for the synthesis of α,β-unsaturated ketones; (**b**)Transition-metal catalyzed α-alkenylation of ketones; (**c**) Iron-catalyzed acceptorless dehydrogenative coupling of alcohols and ketones.

α,β-unsaturated ketones are ubiquitous in various pharmaceutically important plants, and are extensively used as life-saving drugs (choleretic, antiulcer and muco-protective) [1]. Such compounds have wide applications as food additives, pesticides, solar-creams, as well as in materials science [2,3]. Owing to its great demand, synthesis of α,β-unsaturated ketones attracts potential attention in chemical research. In general, Aldol condensation of aldehydes with a suitable ketone as the coupling partner is utilized for such α,β-unsaturated ketones in combination with a stoichiometric amount of a strong base [4,5,6,7]. However, such practice not only produces stoichiometric amounts of waste, also raises major concerns about the uses of expensive and highly susceptible aldehydes (Scheme 1a). Though, the Rh-catalyzed protocol having a combination of ketones with internal alkynes was reported for such α,β-unsaturated ketones; this required higher catalyst loading and stoichiometric amounts of ligands for successful transformations [4,5,6,7]. Since the last decade, the utilization of biomass-derived renewable alcohols has attained significant attention in catalytic research following hydrogen-borrowing strategy. Alcohols are radially available, earth abundant, easy to store, and often generate water as their sole byproduct, when used as one of the coupling partners in organic transformations [8,9,10,11,12,13,14,15]. Therefore, recently, a series of transition metal-based catalysts, such as Ru [16], Pd [17,18], Pt [19], Au [20], Ti [21], Nb [22] and Cr [23] were established for the synthesis of α,β-unsaturated ketones using alcohols. Importantly, a couple of heterogeneous catalysts based on CeO_2_ [24], Bi_2_WO_6_ [25] and mesoporous Pd-nanoparticles [26], were also reported for such alkenylation of ketones with alcohols (Scheme 1b).

Recently, much effort has been devoted towards the replacement of the precious-metal-based catalysts with earth abundant and non-precious metals, such as, Fe-, Mn-, Ni-, Co-, etc., for such transformations using alcohols. In this direction, only one report using a manganese-based complex is known (Scheme 1b). 

However, such Mn-based catalyst bearing the PNP-pincer ligand is the key for successful transformations and selectivity [27]. Importantly, such PNP-ligands require a multistep synthesis process, and are quite a lot more expensive than the non-precious metals. Therefore, there is still a need to develop a simple, efficient and cost-economic catalyst system for the synthesis of α,β-unsaturated ketones. 

Recently, we have started a program for the applications of non-precious, metal-based catalysts for sustainable organic transformations using nickel [28,29,30], manganese [31] and iron-based-catalysts. Most recently, we successfully developed the acceptor-less dehydrogenative coupling of alcohols with alkyl *N*-heteroaromatics using a simple iron-catalyst [32,33]. Iron is the most earth-abundant, biocompatible, and exists in variable oxidation states. Therefore, application of Fe-based catalysts in organic synthesis attract significant attention [34,35,36,37,38,39,40]. Interestingly, iron catalysts were extensively used in cross-coupling reactions, [41,42,43,44,45] hydrogenations and transfer hydrogenations, [46,47,48,49], hydrosilylations [50,51,52], as well as in hydroboration processes [53,54]. However, the application of a Knölker-type complex for the alkylation and methylation of ketones and alcohols is noteworthy [55,56,57,58,59]. Nevertheless, Knölker-type iron complexes require a tedious synthesis procedure and are expensive in nature. Therefore, the recent surge is to develop a simple and inexpensive iron catalyst system in combination with a nitrogen ligand for the dehydrogenative coupling of alcohols with ketones to α, β-unsaturated ketones. To the best of our knowledge, to date, no such iron-catalyzed dehydrogenative coupling of alcohols and ketones to α,β-unsaturated ketones is known (Scheme 1c).

## 2. Results and Discussions

In our initial investigation for the α-alkenylation of ketones, we choose α-tetralone (**1a**) and 4-methoxy benzyl alcohol (**2a**) as our model substrates. Iron catalysts having oxidation states 0, II and III were examined in combination with 1,10 phenanthroline **L1** (6 mol%), *t*-BuONa (0.25 equiv.) in toluene at 110 °C equipped with a nitrogen balloon. To our delight, iron (II) acetate resulted in the formation of α,β-unsaturated ketone **3a** in 75% isolated yield with excellent selectivity (>25:1, Table 1, entry 1 and SI Table S1). Thereafter, a variety of aromatic and aliphatic nitrogen-based ligands, along with triphenyl phosphine **L2**–**L6**, were screened, and no increment in product yield was observed (Table 1, entries 4–8 and SI Table S2). Nevertheless, the application of other alkoxide, carbonate and phosphate bases proved less efficient for such transformation (Table 1, entries 9, 10 and SI Table S3). 

Thereafter, the replacement of toluene by different nonpolar and polar solvents (*p*-xylene, 1,4-dioxane, DMA and *t*-amyl alcohol) resulted in up to 45% conversion to **3a** (Table 1, entry 11 and SI Table S4). α-alkenylation could also be performed using lower catalyst loading (2.5 mol%), however, the product conversion decreases to 53% with (>26:1) selectivity (Table 1, entry 12). Notably, this reaction could also be performed at lower temperature, and resulted up to 58% conversion to the desired product (Table 1, entry 13 and SI Table S6). Catalytic alkenylation for 9 h gave only moderate product conversion along with unreacted starting substrate (Table 1, entry 14 and SI Table S7). Control experiments in the absence of catalyst, ligand and base establish the potential role of the individual component for the α-alkenylation (Table 1, entries 15, 16 and SI Scheme S4 and Scheme S5). Notably, we have detected benzaldehyde **2a**’ and the alkylated product **3a**’ in the gas chromatography–mass spectrometry (GC-MS) analysis of the crude reaction mixture.

**Scheme 2:**
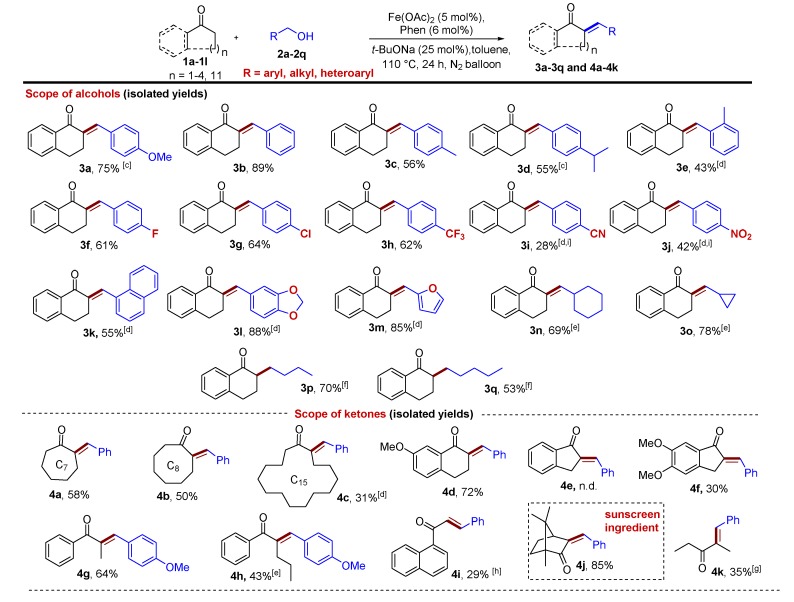
Substrate scope for the Fe-catalyzed α-alkenylation of ketones and alcohols [a, b]. Reaction conditions: [a] Unless specified otherwise, the reaction was carried out with **1a** (0.375 mmol), **2a** (0.25 mmol), Fe(OAc)_2_ (0.0125 mmol), ligand (0.015 mmol) and *t*-BuONa (0.0625 mmol) under an N_2_ balloon at 110 °C (oil bath) in toluene (1.0 mL) for 24 h in a Schlenk tube. [b] The isolated yield reported. [c] The reaction time 12 h. [d] Here, *t*-BuONa (0.125 mmol) was used. [e] In this case, *t*-BuONa (0.25 mmol) was used. [f] For this, α-tetralone (0.25 mmol), alcohols (0.5 mmol) and *t*-BuONa (0.25 mmol) were used. [g] In this case, benzyl alcohol (0.25 mmol), 3-pentanone (0.75 mmol) and *t*-BuONa (0.125 mmol) were used. [h] GC-MS yield. [i] Protonic nuclear magnetic resonance (^1^H-NMR) yield reported.

Next, the optimized conditions of Table 1 were successfully employed for the α-alkenylation using a series of primary alcohols and ketones, and resulted in moderate to excellent yields (Scheme 2). Initially, we explored the reactivity of electronically different aryl, heteroaryl and alkyl alcohols with α-tetralone for the α-alkenylation process. Interestingly, benzyl alcohols, as well as electron-rich *p*-methyl and *p*-*iso*-propyl-substituted benzyl alcohols yielded the α,β-unsaturated ketones **3b**–**3d** in 55–89% isolated yields (Scheme 2). Importantly, sterically hindered *o*-methyl benzyl alcohol **2e** and α-naphthyl methanol **2k** participated efficiently, and resulted the desired products **3e** and **3k** in moderate yields (Scheme 2). To our delight, F, and Cl-substituted benzyl-alcohols were well tolerated under the reaction conditions, and yielded up to 64% of product (**3f** and **3g**), and we did not observe any de-halogenated product.

Notably, benzyl-alcohols bearing strong electron withdrawing groups, such as, CF_3_, CN and NO_2_, also efficiently participated for the α-alkenylation reaction, and the desired products **3h**–**3j** were obtained in up to 62% yield (Scheme 2). More interestingly, heteroaryl alcohols, such as 1,3-dioxolone-substituted benzyl alcohol **2l** and 2-furfurylmethanol **2m** efficiently reacted with α-tetralone to **3l**–**3m** in excellent yields (85–88%, Scheme 2). Thereafter, we explored the reactivity with more challenging alkyl alcohols, such as, cyclohexyl-methanol **2n** and cyclopropyl-methanol **2o**, with **1a**, and the desired products **3n** and **3o** were obtained in 69–78% yield (Scheme 2). However, in the case of acyclic alkyl alcohols, such as, *n*-butanol **2p** and *n*-pentanol **2q**, we observed the formation of the reduced products **3p** and **3q** in up to 70% yield (Scheme 2).

Thereafter, to establish the generality of the α-alkenylation process, we further explored the reactivity of more challenging cyclic and acyclic ketones with alcohols (Scheme 2). To our delight C7, C8 and C15 membered cyclic ketones underwent the reaction smoothly and afforded selective mono-benzylated products **4a**–**4c** in up to 58% isolated yields. Again, 7-methoxy tetralone **1e** and 5,6 dimethoxy indanone **1g** efficiently converted to the α,β-unsaturated ketones **4d** and **4f** in up to 72% isolated yields. Nevertheless, in case of indanone **1f**, we did not observe any desired product (Scheme 2). Notably, propiophenone **1h**, valerophenone **1i** and 1-acetyl naphthalene **1j** reacted smoothly with 4-methoxy benzyl alcohol **2a** and benzyl alcohol **2b**, and transformed into the desired α,β-unsaturated ketones **4g**–**4i** in moderate to good yields (Scheme 2). Interestingly, the reaction of more challenging, 3-pentanone **1l** was sluggish, and the product **4k** was obtained in acceptable yield. Additionally, we attempted the reaction of benzyl alcohol with more challenging and highly sterically hindered camphor **1k.** The desired α, β-unsaturated ketone **4j**, extensively used as a sunscreen ingredient, was obtained in an 85% yield (Scheme 2).

Notably, the established catalytic protocol is tolerant to a series of cyclic and acyclic ketones, as well as benzyl alcohols having sensitive functional moieties, such as, nitro, nitrile, trifluoromethyl, fluoro, chloro, 1,3-dioxolone, alkyl and alkoxy, including furan functionality. As a highlight, we have demonstrated the synthesis of the sunscreen ingredient **4j**.

**Scheme 3:**
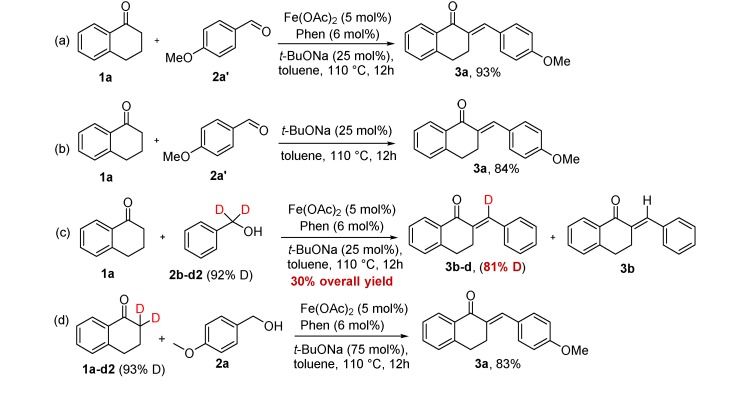
Preliminary mechanistic study for the α-alkenylation of ketone.

After having successfully explored the reactivity of various alcohols and ketones for the alkenylation process, next we focused upon the preliminary mechanistic investigations. To establish the participation of 4-methoxybenzaldehyde **2a‘**, an in-situ ^1^H-NMR study was performed, and we detected the formation of aldehyde in ^1^H-NMR, as well as in the GC-MS analysis of the crude reaction mixture. Again, to demonstrate the potential role of Fe-catalyst for the hydrogen-borrowing process, as well as for the formation of the C–C bond, the reaction of α-tetralone **1a** and 4-methoxy benzyl-aldehyde **2a‘** in the presence and absence of our Fe-catalyst was performed. The desired product was obtained in up to 93% yield (Scheme 3a,b). Next, to make evident the involvement of the benzylic C–H/D bond for the alkenylation process, a deuterium labeling experiment was carried out using deuterated benzyl-alcohol **2b–d2** (**92% D**) with α-tetralone **1a**, and **3b–d** was obtained having 81% deuterium incorporation at the vinyl position (Scheme 3c). However, when the reaction of **1a–d2** (**93% D**) with **2a** was performed, we did not observe any deuterium incorporation in the product **3a** (Scheme 3d). These experiments are in agreement for the micro-reversible transformation in the hydrogen-borrowing process.

**Figure 1:**
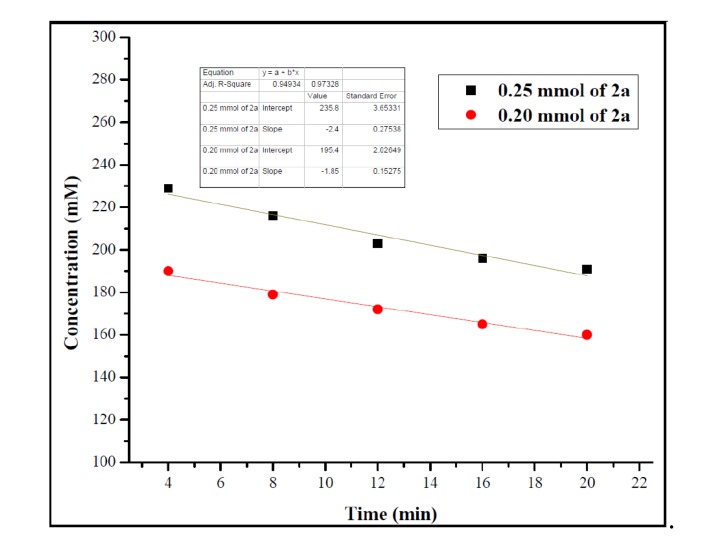
Plot of [**2a**] vs time.

**Figure 2:**
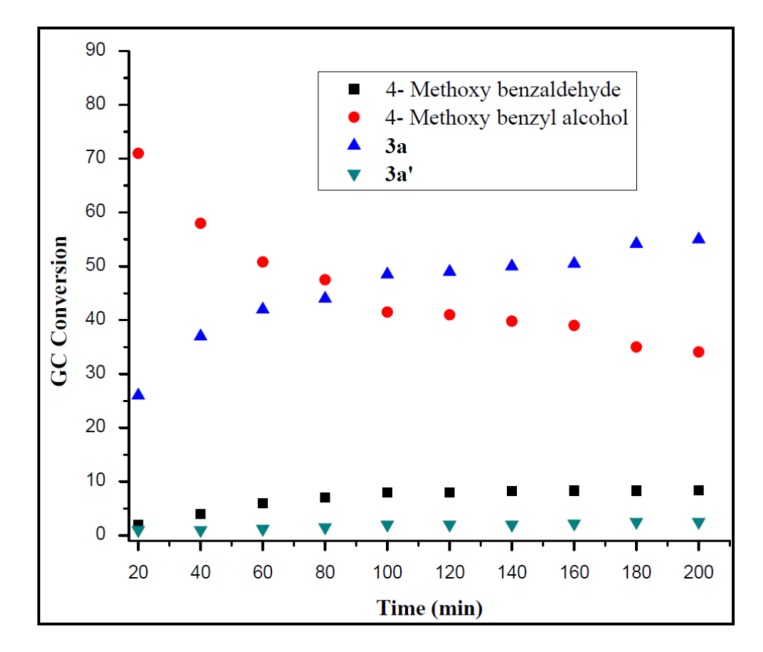
Reaction profile.

Next, to determine the rate and order of the reaction, we performed two different sets of experiments having a variable concentration of ketones and alcohols. The reaction follows first order kinetics with respect to alcohol, considering the steady state approximation for ketone (Figure 1 and SI Scheme S2). Further, we have studied the time-dependent reaction profile for the model reaction of Table 1, and revealed that the concentration of aldehyde is almost constant during the progress of the reaction, whereas, the formation of the reduced product **3a‘** increases with prolonged time (Figure 2). Nevertheless, as expected, hydrogen gas is generated during the dehydrogenation process, so for the quantitative determination of the hydrogen gas, we choose the model reaction of Table 1, and it was connected to the gas burette, as shown in Scheme S3. Gratifyingly, the quantitative determination of hydrogen gas produced in the alkenylation process was calculated (SI Scheme S3).

**Scheme 4:**
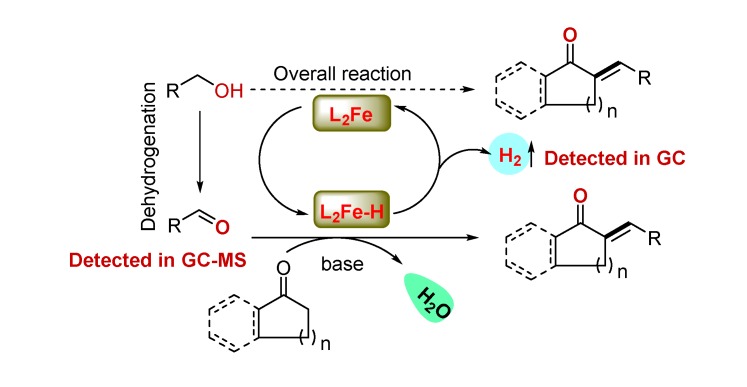
Plausible mechanistic cycle.

Based on these experimental findings, herein we proposed a plausible mechanistic cycle for the *α*-alkenylation of ketones presented in Scheme 4. Primarily, Fe-catalyzed dehydrogenation of alcohol resulted in the formation of aldehyde, and transient iron-hydride is formed. Next, a base-mediated condensation of aldehyde with ketone affords the desired *α*, *β*-unsaturated ketone, releasing water as the byproduct. During the process, the active iron catalyst regenerated via the release of dihydrogen gas.

## 3. Materials and Methods

### 3.1. General Experimental Details

All solvents and reagents were used, as received from the suppliers. Thin-layer chromatography (TLC) was performed on Merck Kiesel gel 60, F_254_ plates with a layer thickness of 0.25 mm. Column chromatography was performed on silica gel (100–200 mesh) using a gradient of ethyl acetate and hexane as the mobile phase.

Protonic nuclear magnetic resonance (^1^H NMR) spectral data were collected at 400 MHz (JEOL), 500 MHz (Bruker), and caron-13 nuclear magnetic resonance (^13^C NMR) values were recorded at 100 MHz. ^1^H NMR spectral data are given as chemical shifts in ppm followed by multiplicity (*s*- singlet; *d*- doublet; *t*- triplet; *q*- quartet; *m*- multiplet), the number of protons, and the coupling constants. ^13^C NMR chemical shifts are expressed in ppm. High resolution mass spectrometry (HRMS) electrospray ionization (ESI) spectral data were collected using a Bruker High Resolution Mass Spectrometer. GC-MS was recorded using Agilent Gas Chromatography Mass Spectrometry. Elemental analysis data were recorded using the Vario Micro Cube elemental analyzer. All the reactions were performed in a closed system using a Schlenk tube. Fe(OAc)_2_ was purchased from TCI Chemicals (India) Pvt. Ltd. (Purity->90%, CAS No: 3094-87-9, Product Number: I0765). 1, 10-Phenanthroline was purchased from Sigma-Aldrich ((Assay- >99%; CAS Number- 66-71-7; EC Number 200-629-2; Pack Size- 131377-25G). Sodium *tert*-butoxide was purchased from Avra Synthesis Pvt. Ltd., India. (Purity-98%, CAS No: **865-48-5**, Catalog No- ASS2615).

### 3.2. General Procedure for Iron-Catalyzed Alkenylation of Ketones with Alcohols:

In a 15 mL oven-dried Schlenk tube, alcohols (0.25 mmol), *t*-BuONa (0.0625 mmol), Fe(OAc)_2_ (0.0125 mmol), phen (0.015 mmol) and ketones (0.375 mmol, 1.5 equiv.) were added followed by toluene 1.0 mL under an atmosphere of an N_2_ balloon, and the reaction mixture was refluxed at 110 °C for 24 h in a closed system. The reaction mixture was cooled to room temperature, and 3.0 mL of ethyl acetate was added and concentrated in vacuo. The residue was purified by column chromatography, using a gradient of hexane and ethyl acetate (eluent system) to afford the pure product.

*(E)-2-(4-methoxybenzylidene)-3,4-dihydronaphthalen-1(2H)-one* (Scheme 2, **3a**) [27], Yellow solid (50 mg, 75% yield); ^1^H NMR (400 MHz, CDCl_3_) *δ* 8.05 (d, *J* = 8.7 Hz, 1H), 7.78 (s, 1H), 7.43–7.34 (m, 3H), 7.29 (t, *J* = 7.5 Hz, 1H), 7.17 (d, *J* = 8.8 Hz, 1H), 6.88 (d, *J* = 8.8 Hz, 2H), 3.78 (s, 3H), 3.09–3.05 (m, 2H), 2.89–2.86 (m, 2H); ^13^C NMR (100 MHz, CDCl_3_) *δ* 186.9, 158.9, 142.0, 135.7, 132.6, 132.5, 132.1, 130.7, 127.4, 127.1, 127.0, 126.0, 112.9, 54.3, 27.8, 26.2.

*(E)-2-benzylidene-3,4-dihydronaphthalen-1(2H)-one (*Scheme 2, **3b***)* [27], Colorless solid (52 mg, 89% yield); ^1^H NMR (500 MHz, CDCl_3_) *δ* 8.06 (dd, *J* = 7.8, 0.9 Hz, 1H), 7.80 (s, 1H), 7.43–7.40 (m, 1H), 7.39–7.31 (m, 4H), 7.29–7.26 (m, 2H), 7.19–7.15 (m, 1H), 3.07–3.04 (m, 2H), 2.88–2.83 (m, 2H); ^13^C NMR (125 MHz, CDCl_3_) *δ* 186.9, 142.2, 135.6, 134.8, 134.5, 132.5, 132.2, 128.9, 127.5, 127.4, 127.2, 127.1, 126.0, 27.9, 26.2.

*(E)-2-(4-methylbenzylidene)-3,4-dihydronaphthalen-1(2H)-one* (Scheme 2, **3c**) [27], Colorless solid (35 mg, 56% yield); ^1^H NMR (500 MHz, CDCl_3_) *δ* 8.06 (d, *J* = 7.8 Hz, 1H), 7.78 (s, 1H), 7.43–7.40 (m, 1H), 7.29 (dd, *J* = 7.3, 5.2 Hz, 2H), 7.19–7.15 (m, 4H), 3.08–3.05 (m, 2H), 2.89–2.86 (m, 2H), 2.32 (s, 3H); ^13^C NMR (125 MHz, CDCl_3_) *δ* 187.9, 143.2, 138.8, 136.8, 134.7, 133.6, 133.2, 133.0, 130.0, 129.2, 128.2, 128.1, 127.0, 28.9, 27.2, 21.4.

*(E)-2-(4-isopropylbenzylidene)-3,4-dihydronaphthalen-1(2H)-one* (Scheme 2, **3d**) [27], Yellow oil (40 mg, 55% yield); ^1^H NMR (500 MHz, CDCl_3_) *δ* 8.05 (dd, *J* = 7.8, 1.0 Hz, 1H), 7.79 (s, 1H), 7.42–7.39 (m, 1H), 7.34–7.26 (m, 3H), 7.21 (d, *J* = 8.2 Hz, 2H), 7.17 (d, *J* = 8.1 Hz, 1H), 3.09–3.06 (m, 2H), 2.88–2.85 (m, 3H), 1.20 (d, *J* = 6.9 Hz, 6H); ^13^C NMR (125 MHz, CDCl_3_) *δ* 187.9, 149.7, 143.2, 136.8, 134.7, 133.6, 133.4, 133.2, 130.1, 128.2, 128.1, 127.0, 126.6, 34.0, 28.9, 27.3, 23.9.

*(E)-2-(2-methylbenzylidene)-3,4-dihydronaphthalen-1(2H)-one* (Scheme 2, **3e**) [27], Yellow oil (27 mg, 43% yield); ^1^H NMR (500 MHz, CDCl_3_) *δ* 8.19 (dd, *J* = 7.8, 1.2 Hz, 1H), 7.94 (s, 1H), 7.54–7.51 (m, 1H), 7.40 (t, *J* = 7.1 Hz, 1H), 7.29–7.26 (m, 5H), 3.02–2.99 (m, 2H), 2.98–2.95 (m, 2H), 2.37 (s, 3H); ^13^C NMR (125 MHz, CDCl_3_) *δ* 188.1, 143.6, 137.9, 136.1, 135.8, 135.2, 133.6, 133.4, 130.4, 129.0, 128.6, 128.4, 128.3, 127.2, 125.6, 29.4, 27.4, 20.2.

*(E)-2-(4-fluorobenzylidene)-3,4-dihydronaphthalen-1(2H)-one* (Scheme 2, **3f**) [60], Yellow solid (38 mg, 61% yield); ^1^H NMR (500 MHz, CDCl_3_) *δ* 8.06 (dd, J = 7.8, 0.9 Hz, 1H), 7.76 (s, 1H), 7.45–7.42 (m, 1H), 7.36 (dd, J = 8.5, 5.5 Hz, 2H), 7.31 (t, J = 7.6 Hz, 1H), 7.19 (d, J = 7.3 Hz, 1H), 7.05 (t, J = 8.7 Hz, 2H), 3.06–3.03 (m, 2H), 2.91–2.88 (m, 2H); ^13^C NMR (125 MHz, CDCl_3_) *δ* 186.7, 161.6 (d, J _C-F_ = 249.9 Hz), 142.1, 134.5, 134.3, 132.4, 132.3, 130.9, 130.8, 130.7, 127.2 (d, J _C-F_ = 9.2 Hz), 126.1, 114.6 (d, J _C-F_ = 21.7 Hz), 27.7, 26.1.

*(E)-2-(4-chlorobenzylidene)-3,4-dihydronaphthalen-1(2H)-one* (Scheme 2, **3g**) [60], Yellow solid (44 mg, 64% yield); ^1^H NMR (500 MHz, CDCl_3_) *δ* 8.06 (d, *J* = 7.8 Hz, 1H), 7.73 (s, 1H), 7.44–7.38 (m, 1H), 7.30 (dd, *J* = 6.7, 4.1 Hz, 4H), 7.19 (dd, *J* = 7.5, 4.9 Hz, 2H), 3.04–3.01 (m, 2H), 2.90–2.87 (m, 2H); ^13^C NMR (125 MHz, CDCl_3_) *δ* 186.6, 142.1, 135.0, 134.2, 133.4, 133.3, 132.4, 130.1, 127.7, 127.3, 127.2, 126.1, 114.0, 27.8, 26.2.

*(E)-2-(4-(trifluoromethyl)benzylidene)-3,4-dihydronaphthalen-1(2H)-one* (Scheme 2, **3h**) [61], Yellow solid (47 mg, 62% yield); ^1^H NMR (500 MHz, CDCl_3_) *δ* 8.07 (d, *J* = 7.8 Hz, 1H), 7.77 (s, 1H), 7.60 (d, *J* = 8.2 Hz, 2H), 7.46–7.42 (m, 3H), 7.31 (t, *J* = 7.5 Hz, 1H), 7.19 (d, *J* = 7.7 Hz, 1H), 3.03 (td, *J* = 6.4, 1.6 Hz, 2H), 2.91–2.88 (m, 2H); ^13^C NMR (125 MHz, CDCl_3_) *δ* 186.5, 142.2, 138.4, 136.4, 133.7, 132.5, 132.2, 129.3, 129.0, 128.9, 128.4, 127.3–127.2 (d, *J*_C-F_ = 7.3 Hz), 126.1, 124.4–124.3 (q, *J*_C-F_ = 3.8 Hz), 124.0, 121.9, 27.8, 26.1.

*(E)-4-((1-oxo-3,4-dihydronaphthalen-2(1H)-ylidene)methyl)benzonitrile (*Scheme 2, **3i***)* [62], ^1^H NMR (500 MHz, CDCl_3_) *δ* 8.12 (d, *J* = 7.7 Hz, 1H), 7.83 (s, 1H), 7.69 (d, *J* = 8.3 Hz, 2H), 7.46 (td, *J* = 7.5, 1.5 Hz, 3H), 7.35 (d, *J* = 3.2 Hz, 1H), 7.19 (d, *J* = 3.9 Hz, 1H), 3.10–3.04 (t, 2H), 2.95 (t, *J* = 6.1 Hz, 2H); ^13^C NMR (125 MHz, CDCl_3_) *δ* 187.4, 143.1, 140.5, 138.2, 138.1, 134.1, 133.4, 132.3, 132.2, 128.8, 128.4, 128.3, 127.3, 126.7, 111.8, 111.1, 28.7, 23.3.

*(E)-2-(4-nitrobenzylidene)-3,4-dihydronaphthalen-1(2H)-one (*Scheme 2, **3j***)* [60], ^1^H NMR (500 MHz, CDCl_3_) *δ* 8.30 (d, *J* = 1.9 Hz, 2H), 8.14 (d, *J* = 1.3 Hz, 1H), 7.85 (s, 1H), 7.59–7.55 (m, 3H), 7.40–7.38 (m, 1H), 7.30 (s, 1H), 3.15–3.07 (m, 2H), 3.00–2.96 (m, 2H); ^13^C NMR (125 MHz, CDCl_3_) *δ* 187.3, 147.4, 144.7, 138.6, 137.3, 133.8, 133.5, 132.7, 130.5, 128.8, 127.3, 126.7, 123.8, 28.8, 23.4.

*(E)-2-(naphthalen-1-ylmethylene)-3,4-dihydronaphthalen-1(2H)-one* (Scheme 2, **3k**) [27], Yellow solid (39 mg, 55% yield); ^1^H NMR (500 MHz, CDCl_3_) *δ* 8.30 (s, 1H), 8.14 (dd, *J* = 7.8, 1.1 Hz, 1H), 7.95–7.93 (m, 1H), 7.83–7.79 (m, 2H), 7.49–7.42 (m, 4H), 7.36–7.31 (m, 2H), 7.18 (t, *J* = 3.7 Hz, 1H), 2.93–2.91 (m, 2H), 2.85 (t, *J* = 6.5 Hz, 2H); ^13^C NMR (125 MHz, CDCl_3_) *δ* 186.8, 142.6, 136.5, 133.7, 132.6, 132.5, 132.4, 132.1, 131.0, 127.9, 127.5, 127.4, 127.3, 126.0, 125.8, 125.4, 125.2, 124.1, 123.9, 28.2, 26.7.

*(E)-2-(benzo [d]* [1,3]*dioxol-5-ylmethylene)-3,4-dihydronaphthalen-1(2H)-one* (Scheme 2, **3l**) [27], Yellow solid (61 mg, 88% yield); ^1^H NMR (500 MHz, CDCl_3_) *δ* 8.11 (dd, *J* = 7.8, 0.9 Hz, 1H), 7.79 (s, 1H), 7.49–7.46 (m, 1H), 7.35 (t, *J* = 7.5 Hz, 1H), 7.27–7.23 (m, 1H), 6.99–6.95 (m, 2H), 6.86 (d, *J* = 8.0 Hz, 1H), 6.00 (s, 2H), 3.13–3.11 (m, 2H), 2.96–2.93 (m, 2H); ^13^C NMR (125 MHz, CDCl_3_) *δ* 187.8, 148.0, 147.8, 143.1, 136.7, 134.0, 133.6, 133.2, 129.9, 128.2, 127.0, 125.1, 115.0, 109.8, 108.5, 101.4, 28.8, 27.3.

*(E)-2-(furan-2-ylmethylene)-3,4-dihydronaphthalen-1(2H)-one* (Scheme 2, **3m**) [27], Yellow solid (48 mg, 85% yield); ^1^H NMR (500 MHz, CDCl_3_) *δ* 8.11 (dd, *J* = 7.8, 1.3 Hz, 1H), 7.60–7.56 (m, 2H), 7.50–7.46 (m, 1H), 7.37–7.33 (m, 1H), 7.27 (d, *J* = 7.5 Hz, 1H), 6.71 (d, *J* = 3.5 Hz, 1H), 6.52 (dd, *J* = 3.4, 1.8 Hz, 1H), 3.36–3.31 (m, 2H), 3.03–2.99 (m, 2H); ^13^C NMR (125 MHz, CDCl_3_) *δ* 187.4, 152.5, 144.3, 143.5, 133.6, 133.1, 131.9, 128.1, 127.0, 122.8, 116.6, 115.0, 112.2, 28.4, 26.7.

*(E)-2-(cyclohexylmethylene)-3,4-dihydronaphthalen-1(2H)-one* (Scheme 2, **3n**), Yellow solid (41 mg, 69% yield); ^1^H NMR (500 MHz, CDCl_3_) *δ* 8.02 (dd, *J* = 7.8, 1.0 Hz, 1H), 7.40–7.36 (m, 1H), 7.25 (t, *J* = 7.5 Hz, 1H), 7.16 (d, *J* = 7.5 Hz, 1H), 6.70 (d, *J* = 9.7 Hz, 1H), 2.89–2.86 (m, 2H), 2.74–2.71 (m, 2H), 2.36–2.27 (m, 1H), 1.72–1.68 (m, 2H), 1.63–1.58 (m, 3H), 1.28–1.14 (m, 5H); ^13^C NMR (125 MHz, CDCl_3_) *δ* 186.9, 144.1, 142.7, 132.7, 132.3, 131.9, 127.2, 127.1, 125.8, 36.2, 31.2, 28.3, 24.9, 24.8, 24.6. HRMS (ESI): Calculated for [C_17_H_21_O]^+^ 241.1587; Found 241.1589.

*(E)-2-(cyclopropylmethylene)-3,4-dihydronaphthalen-1(2H)-one* (Scheme 2, **3o**) [63], White solid (39 mg, 78% yield); ^1^H NMR (500 MHz, CDCl_3_) *δ* 8.01 (dd, *J* = 7.8, 1.1 Hz, 1H), 7.39–7.37 (m, 1H), 7.26 (t, *J* = 7.5 Hz, 1H), 7.18 (d, *J* = 7.6 Hz, 1H), 6.27 (d, *J* = 10.9 Hz, 1H), 2.93–2.86 (m, 2H), 2.86–2.83 (m, 2H), 1.67–1.60 (m, 1H), 0.97–0.93 (m, 2H), 0.68–0.65 (m, 2H); ^13^C NMR (125 MHz, CDCl_3_) *δ* 186.7, 145.6, 143.6, 133.7, 132.8, 132.7, 128.2, 128.1, 126.8, 29.0, 25.5, 11.8, 9.0.

*2-butyl-3,4-dihydronaphthalen-1(2H)-one* (Scheme 2, **3p**) [64], Pale yellow liquid (36 mg, 70% yield); ^1^H NMR (500 MHz, CDCl_3_) *δ* 7.96 (dd, *J* = 7.8, 1.0 Hz, 1H), 7.40–7.36 (m, 1H), 7.23 (d, *J* = 7.6 Hz, 1H), 7.16 (d, *J* = 7.6 Hz, 1H), 2.94–2.86 (m, 3H), 2.42–2.38 (m, 1H), 2.20–2.14(m, 1H), 1.90–1.80 (m, 2H), 1.44–1.40 (m, 1H), 1.36–1.28 (m, 3H), 0.85 (t, *J* = 7.0 Hz, 3H); ^13^C NMR (125 MHz, CDCl_3_) *δ* 199.5, 143.0, 132.0, 131.6, 127.6, 126.4, 125.5, 46.5, 28.2, 28.1, 27.3, 27.2, 21.8, 13.0.

*2-pentyl-3,4-dihydronaphthalen-1(2H)-one* (Scheme 2, **3q**) [65], Pale yellow liquid (29 mg, 53% yield); ^1^H NMR (400 MHz, CDCl_3_) *δ* 7.96 (d, *J* = 7.8 Hz, 1H), 7.40–7.36 (m, 1H), 7.23 (t, *J* = 7.5 Hz, 1H), 7.16 (d, *J* = 7.5 Hz, 1H), 2.98–2.84 (m, 3H), 2.44–2.37 (m, 1H), 2.20–2.13 (m, 1H), 1.91–1.78 (m, 2H), 1.35–1.21 (m, 6H), 0.83 (t, *J* = 6.6 Hz, 3H); ^13^C NMR (100 MHz, CDCl_3_) *δ* 199.5, 142.9, 132.0, 131.5, 127.6, 126.4, 125.5, 46.5, 30.9, 28.3, 27.3, 27.1, 25.7, 21.6, 13.1.

*(E)-2-benzylidenecycloheptan-1-one* (Scheme 2, **4a**) [66], Colorless liquid (29 mg, 58% yield); ^1^H NMR (400 MHz, CDCl_3_) *δ* 7.45 (s, 1H), 7.34–7.29 (m, 2H), 7.28–7.22 (m, 3H), 2.66–2.61 (m, 4H), 1.77–1.68 (m, 6H); ^13^C NMR (100 MHz, CDCl_3_) *δ* 205.0, 140.8, 136.1, 135.7, 129.5, 128.5, 43.5, 31.4, 30.0, 27.7, 25.5.

*(E)-2-benzylidenecyclooctanone* (Scheme 2, **4b**) [66], Colorless liquid (27 mg, 50% yield); ^1^H NMR (500 MHz, CDCl_3_) *δ* 7.40 (s, 1H), 7.31 (dd, *J* = 10.4, 2.8 Hz, 4H), 7.28–7.24 (m, 1H), 2.76–2.74 (m, 2H), 2.65 (dd, *J* = 7.3, 5.5 Hz, 2H), 1.81–1.76 (m, 2H), 1.71–1.66 (m, 2H), 1.60–1.55 (m, 2H), 1.48–1.43 (m, 2H); ^13^C NMR (125 MHz, CDCl_3_) *δ* 206.6, 139.5, 135.5, 135.1, 128.6, 127.4, 127.4, 38.3, 29.1, 28.6, 25.5, 24.9, 24.5.

*(E)-2-benzylidenecyclopentadecanone* (Scheme 2, **4c**), Colorless liquid (24 mg, 31% yield); ^1^H NMR (500 MHz, CDCl_3_) *δ* 7.43–7.41 (m, 5H), 7.37–7.34 (m, 1H), 2.84–2.81 (m, 2H), 2.65 (t, *J* = 7.0 Hz, 2H), 2.44 (t, *J* = 6.7 Hz, 1H), 1.78–1.75 (m, 2H), 1.49 (dd, *J* = 9.1, 6.2 Hz, 2H), 1.39–1.29 (m, 17H); ^13^C NMR (125 MHz, CDCl_3_) *δ* 204.0, 143.3, 137.2, 136.1, 129.2, 128.5, 128.2, 42.1, 37.6, 28.6, 27.8, 27.6, 27.5, 26.8, 26.7, 26.5, 26.4, 26.2, 26.1, 24.3, 23.5. HRMS (ESI): Calculated for [C_22_H_33_O]^+^ 313.2526; Found 241.2528.

*(E)-2-benzylidene-7-methoxy-3,4-dihydronaphthalen-1(2H)-one* (Scheme 2, **4d**) [66], Yellow solid (48 mg, 72% yield); ^1^H NMR (500 MHz, CDCl_3_) *δ* 7.89 (s, 1H), 7.66 (d, *J* = 2.8 Hz, 1H), 7.48–7.43 (m, 4H), 7.40–7.37 (m, 1H), 7.19 (d, *J* = 8.3 Hz, 1H), 7.10 (dd, *J* = 8.3, 2.8 Hz, 1H), 3.90 (s, 3H), 3.15–3.13 (m, 2H), 2.93–2.42 (m, 2H); ^13^C NMR (125 MHz, CDCl_3_) *δ* 188.0, 158.9, 136.9, 136.2, 136.1, 135.7, 134.5, 130.0, 129.6, 128.7, 128.6, 121.7, 110.5, 55.7, 28.2, 27.6.

*(E)-2-benzylidene-5,6-dimethoxy-2,3-dihydro-1H-inden-1-one* (Scheme 2, **4f**) [67], Yellow solid (21 mg, 30% yield); ^1^H NMR (500 MHz, CDCl_3_) *δ* 7.69 (d, *J* = 7.3 Hz, 2H), 7.63 (t, *J* = 1.8 Hz, 1H), 7.48 (t, *J* = 7.5 Hz, 2H), 7.42 (t, *J* = 2.0 Hz, 1H), 7.38 (s, 1H), 7.02 (s, 1H), 4.03 (s, 3H), 4.01 (d, *J* = 1.6 Hz, 2H), 3.98 (s, 3H); ^13^C NMR (125 MHz, CDCl_3_) *δ* 206.5, 155.6, 148.9, 141.8, 139.8, 139.7, 135.4, 132.4, 128.9, 128.8, 128.0, 115.0, 107.4, 56.2, 56.1, 31.9.

*(E)-3-(4-methoxyphenyl)-2-methyl-1-phenylprop-2-en-1-one* (Scheme 2, **4g**) [27], Colorless oil (40 mg, 64% yield); ^1^H NMR (500 MHz, CDCl_3_) *δ* 7.74 (dd, *J* = 8.2, 1.3 Hz, 2H), 7.57–7.54 (m, 1H), 7.49–7.46 (m, 2H), 7.44–7.42 (m, 2H), 7.18 (s, 1H), 6.97–6.95 (m, 2H), 3.87 (s, 3H), 2.31 (d, *J* = 1.3 Hz, 3H); ^13^C NMR (125 MHz, CDCl_3_) *δ* 199.6, 160.0, 142.6, 139.0, 134.9, 131.6, 131.4, 129.4, 128.4, 128.1, 115.0, 114.0, 55.4, 14.4.

*(E)-2-(4-methoxybenzylidene)-1-phenylpentan-1-one* (Scheme 2, **4h**) [68], Colorless oil liquid. (26 mg, 43% yield); ^1^H NMR (500 MHz, CDCl_3_) *δ* 7.77 (dd, *J* = 8.3, 1.3 Hz, 2H), 7.59–7.53 (m, 1H), 7.47 (dd, *J* = 9.7, 4.0 Hz, 2H), 7.39–7.33 (m, 3H), 6.95 (d, *J* = 8.8 Hz, 2H), 3.87 (s, 3H), 2.78–2.74 (m, 2H), 1.67–1.62 (m, 2H), 1.05 (t, *J* = 7.4 Hz, 3H); ^13^C NMR (125 MHz, CDCl_3_) *δ* 199.5, 159.9, 141.4, 140.4, 139.1, 133.2, 131.0, 129.5, 128.5, 128.2, 114.0, 55.3, 29.6, 22.0, 14.4.

*(1S,4S)-3-(benzylidene)-1,7,7-trimethylbicyclo [2.2.1]heptan-2-one* (Scheme 2, **4j**) [69], Colorless liquid (51 mg, 85% yield); ^1^H NMR (500 MHz, CDCl_3_) *δ* 7.51–7.50 (m, 2H), 7.43–7.40 (m, 2H), 7.38–7.34 (m, 1H), 7.27 (s, 1H), 3.14 (d, *J* = 4.2 Hz, 1H), 2.24–2.18 (m, 1H), 1.82–1.79 (m, 1H), 1.63–1.53 (m, 2H), 1.06 (s, 3H), 1.03 (s, 3H), 0.83 (s, 3H); ^13^C NMR (125 MHz, CDCl_3_) *δ* 208.2, 142.1, 135.7, 129.8, 128.7, 128.6, 127.5, 57.1, 49.2, 46.7, 30.7, 26.0, 20.6, 18.3, 9.3.

*2-methyl-1-phenylpent-1-en-3-one* (Scheme 2, **4k**) [70], Colorless liquid (15 mg, 35% yield); ^1^H NMR (500 MHz, CDCl_3_) *δ* 7.46 (d, *J* = 1.2 Hz, 1H), 7.34 (d, *J* = 4.4 Hz, 3H), 7.28 (d, *J* = 4.1 Hz, 1H), 7.19 (s, 1H), 2.78 (q, *J* = 7.3 Hz, 2H), 2.00 (s, 3H), 1.11 (t, *J* = 7.3 Hz, 3H); ^13^C NMR (125 MHz, CDCl_3_) *δ* 203.0, 138.2, 137.2, 136.1, 129.7,128.4, 128.4, 30.8, 13.2, 8.9.

## 4. Conclusions

In conclusion, we have established a simple, cost-efficient and commercially available iron catalyst system for the dehydrogenative coupling of alcohols and ketones to *α*, *β*-unsaturated ketone. The process is highly selective (>25:1), and a variety of benzyl alcohols bearing nitro, nitrile, trifluoro-methyl fluoro, chloro and 1,3-dioxolone-functionalities, 2-furfurylmethanol, as well as cyclic and acyclic alkyl alcohols, were well tolerated under the optimized reaction conditions. Preliminary mechanistic investigations establish the hydrogen-borrowing process. As a highlight, we have demonstrated the synthesis of a sunscreen ingredient **4j** (Scheme 2).

## Figures and Tables

**Table 1:**
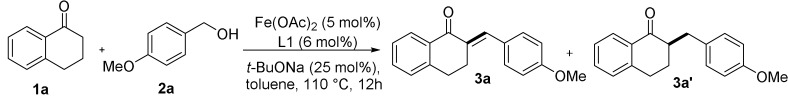
Optimization table for Fe-catalyzed α-alkenylation of ketone [a, b, c].

Entry	Deviations from the above	Conv. 3a (%) [b]	3a/3a’
1	-	**77(75)**	**>25:1**
2	Fe(acac)_3_	68	17:1
3	Fe_2_(CO)_9_	64	16:1
4	**L2** instead of **L1**	68	17:1
5	**L3** instead of **L1**	54	>10:1
6	**L4** instead of **L1**	67	>11:1
7	**L5** instead of **L1**	70	4:1
8	**L6** instead of **L1**	60	2:1
9	*t*-BuOK	58	2:1
10	Na_2_CO_3_, K_2_CO_3_, Cs_2_CO_3_,	12–30	-
11	*p*-xylene, 1,4-dioxane, *t*-amyl-alcohol	25–45	>10
12	Fe(OAc)_2_ (2.5 mol%), **L1** (3.0 mol%)	53	>26:1
13	Reaction at 80 °C	58	-
14	9 h reaction time	62	>31:1
15	no Fe(OAc)_2_, no ligand	20	-
16	No base	0	0

Reaction conditions: [a] Unless specified otherwise, the reaction was carried out with **1a** (0.375 mmol), **2a** (0.25 mmol), Fe-cat. (0.0125 mmol), ligand (0.015 mmol), and *t*-BuONa (0.0625 mmol) under an N_2_ balloon at 110 °C (oil bath) in toluene (1.0 mL) for 12 h in a Schlenk tube. [b] Conversion was determined by gas chromatography–mass spectrometry (GC-MS) (isolated yield in parentheses). [c] = tri-phenyl phosphine (10 mol%) was used. **L1** = 1,10-phenanthroline, **L2** = 2,9-dimethyl-1,10-phenanthroline, **L3** = 2,2′-biquinoline, **L4** = 2,2′-bipyridine, **L5** = triphenyl-phosphine. **L6** = tetramethylethylenediamine (TMEDA).

## Supplementary Materials

The following are available online, 1H-NMR, 13C-NMR, HRMS data and deuterium labeling experiments.
