# Supplementary material for: A Simple Iron-Catalyst for Alkenylation of Ketones Using Primary Alcohols
Source: Molecules. 2020 Mar 30;25(7):1590. doi: 10.3390/molecules25071590 (PMC7181299; doi:10.3390/molecules25071590)
Supplement: Supplementary file 1 [file molecules-25-01590-s001.pdf]

A Simple Iron-catalyst for Alkenylation of Ketones Using Primary Alcohols

Motahar Sk, Ashish Kumar, Jagadish Das, and Debasis Banerjee*

Department of Chemistry
Laboratory of Catalysis and Organic Synthesis
Indian Institute of Technology Roorkee, Roorkee-247667, India
E-mail: debasis.banerjee@cy.iitr.ac.in

Table of Contents

Alkenylation of ketones with alcohols S2

^{1}H NMR and ^{13}C NMR Spectra for selected compounds S9

[1.1] Alkenylation of ketones with alcohols:

Table S1: Screening of catalysts [a]

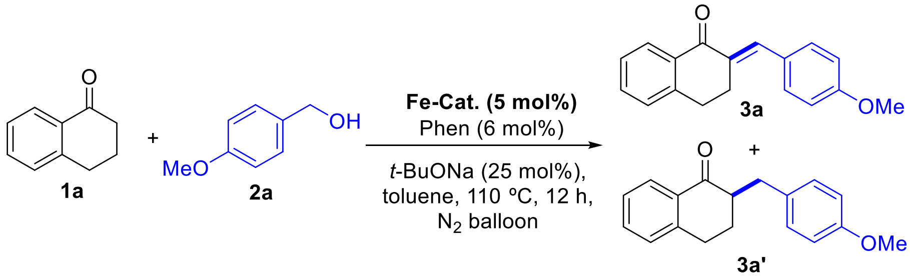

Entry	Fe-Catalyst	GC-MS Conversion **3a** (%)	GC-MS Conversion **3a'** (%)
**1**[b]	**Fe(OAc)$_2$**	**77(75)**	**3**
2	Fe(acac)$_3$	68	4
3	Fe$_2$(CO)$_9$	64	4
4	Fe(OAc)$_2$ (2.5 mol%), Phen (3 mol%)	53	2
5	No Catalyst, No Ligand	20	-

Reaction conditions: [a] *p*-methoxy benzyl alcohol (0.25 mmol), *α*-tetralone (0.375 mmol), **Fe-catalyst (5 mol%)**, Phen (6 mol%), *t*-BuONa (0.0625 mmol), toluene (1.0 mL), Schlenk tube under nitrogen atmosphere, 110 °C oil bath, 12 h reaction time. [b] Isolated yield.

Table S2: Screening of ligands[a]

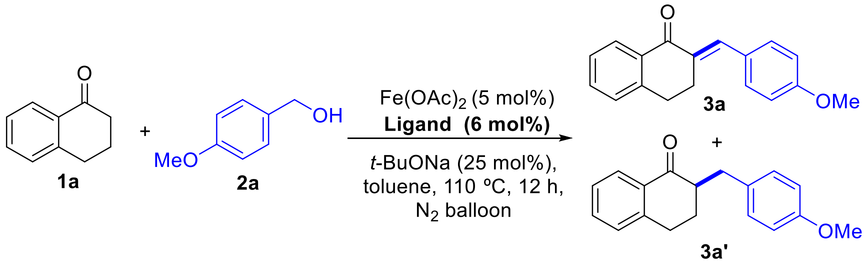

Entry	**Ligand**	GC-MS Conversion **3a** (%)	GC-MS Conversion **3a'** (%)
**1**[b]	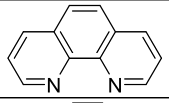 **L1**	**77(75)**	**3**
2	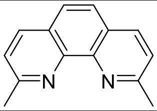 **L2**	68	4
3	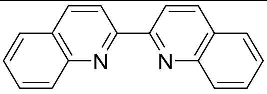 **L3**	54	5
4	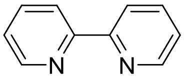 **L4**	67	6
5	PPh$_3$ **(L5)**	70	17
6	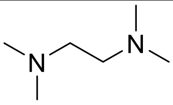 **L6**	60	30
7	No Ligand	43	2

Reaction conditions: [a] *p*-methoxy benzyl alcohol (0.25 mmol), *α*-tetralone (0.375 mmol), $Fe(OAc)_2$ (5 mol%), **Ligand (6 mol%),** *t*-BuONa (0.0625 mmol), toluene (1.0 mL), Schlenk tube under nitrogen atmosphere, 110 °C oil bath, 12 h reaction time. PPh_3 (10 mol%) was used. [b] Isolated yield.

Table S3: Screening of base [a]

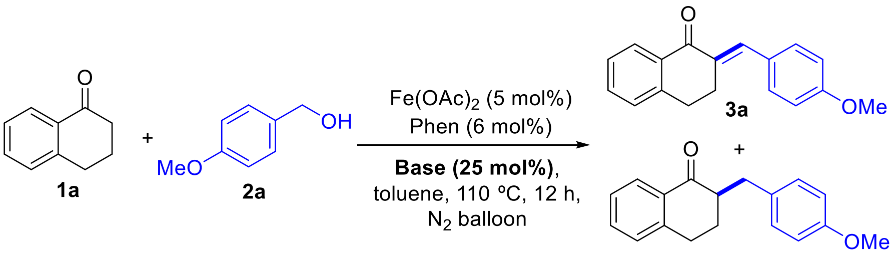

Entry	**Base**	GC-MS Conversion **3a** (%)	GC-MS Conversion **3a'** (%)
**1**[b]	***t*-BuONa**	**77(75)**	**3**
2	*t*-BuOK	58	30
3	Na_2CO_3	25	<1
4	K_2CO_3	30	<1
5	Cs_2CO_3	12	<1
6	K_3PO_4	7	0
7	No Base	0	0

Reaction conditions: [a] *p*-methoxy benzyl alcohol (0.25 mmol), *α*-tetralone (0.375 mmol), $Fe(OAc)_2$ (5 mol%), Phen (6 mol%), **base** (0.0625 mmol), toluene (1.0 mL), Schlenk tube under nitrogen atmosphere, 110 °C oil bath, 12 h reaction time. [b] Isolated yield.

Table S4: Screening of solvents [a]

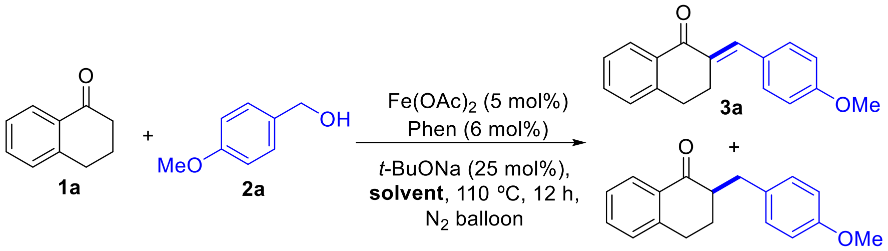

Entry	**Solvent**	GC-MS Conversion **3a** (%)	GC-MS Conversion **3a'** (%)
**1[b]**	**toluene**	**77(75)**	**3**
2	*p*-xylene	45	5
3	1,4-dioxane	33	2
4	DMA	8	0
5	*t*-amylalcohol	25	16

Reaction condition: [a] *p*-methoxy benzyl alcohol (0.25 mmol), *α*-tetralone (0.375 mmol), $Fe(OAc)_2$ (5 mol%), Phen (6 mol%), *t*-BuONa (0.0625 mmol), **solvent** (1.0 mL), Schlenk tube under nitrogen atmosphere, 110 °C oil bath, 12 h reaction time. [b] Isolated yield.

Table S5: Screening of ketone equivalents [a]

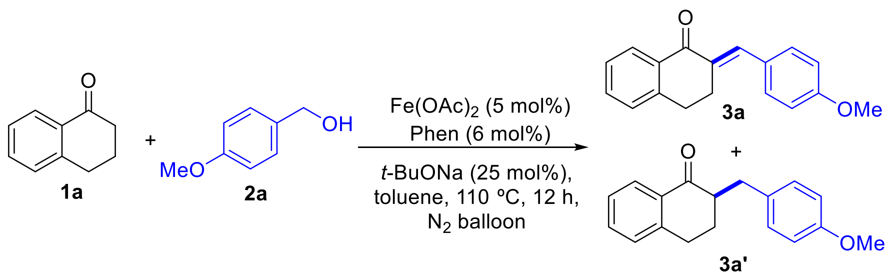

Entry	***α*-tetralone (X equiv.)**	GC-MS Conversion **3a** (%)	GC-MS Conversion **3a'** (%)
1	1.0	54	4
**2**[b]	**1.5**	**77(75)**	**3**

Reaction condition: [a] p-methoxy benzyl alcohol (0.25 mmol), ***α*-tetralone (0.25 mmol, 0.375 mmol)**, $Fe(OAc)_2$ (5 mol%), Phen (6 mol%), *t*-BuONa (0.0625 mmol), toluene (1.0 mL), Schlenk tube under nitrogen atmosphere, 110 °C oil bath, 12 h reaction time. [b] Isolated yield (average of two run).

Table S6: Screening of temperature [a]

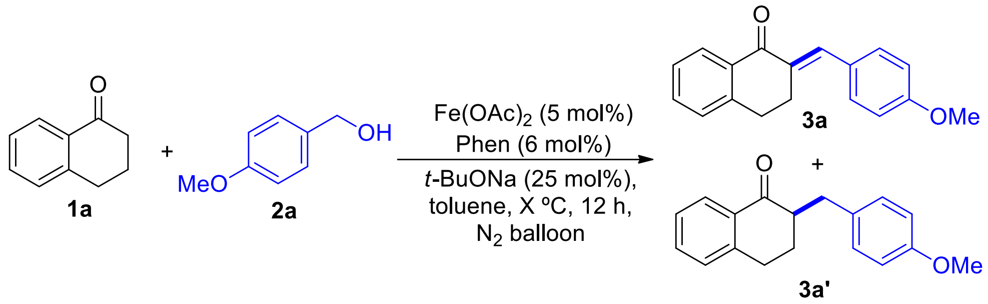

Entry	**Temperature (X ^{0}C)**	GC-MS Conversion **3a** (%)	GC-MS Conversion **3a'** (%)
**1**	**110**	**77(75)**[b]	**3**
2	80	58	-
3	50	9	-

Reaction conditions: [a] *p*-methoxy benzyl alcohol (0.25 mmol), *α*-tetralone (0.375 mmol), $Fe(OAc)_2$ (5 mol%), Phen (6 mol%), NaO^tBu (0.0625 mmol), toluene (1.0 mL), Schlenk tube under nitrogen atmosphere, **X** °C oil bath, 12 h reaction time. [b] Isolated yield.

Table S7: Screening of time [a]

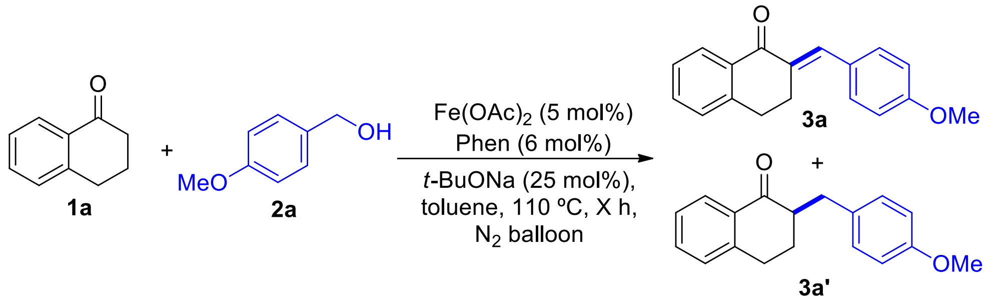

Entry	**Time (X h)**	GC-MS Conversion **3a** (%)	GC-MS Conversion **3a'** (%)
**1**	**12**	**77(75)[b]**	**3**
2	9	62	2
3	6	45	4

Reaction conditions: [a] *p*-methoxy benzyl alcohol (0.25 mmol), *α*-tetralone (0.375 mmol), $Fe(OAc)_2$ (5 mol%), Phen (6 mol%), NaO^tBu (0.0625 mmol), toluene (1.0 mL), Schlenk tube under nitrogen atmosphere, 110 ºC oil bath, **X** h reaction time. [b] Isolated yield.

Deuterium Incorporation Experiments:

Scheme S1:

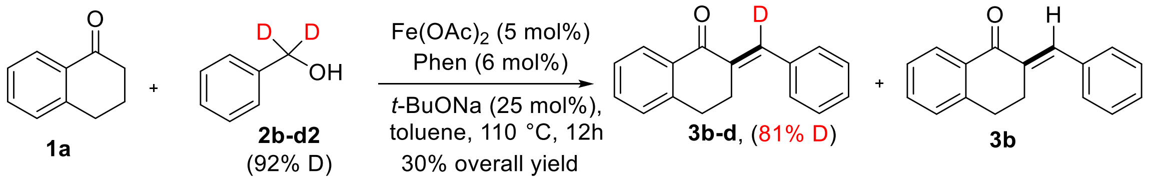

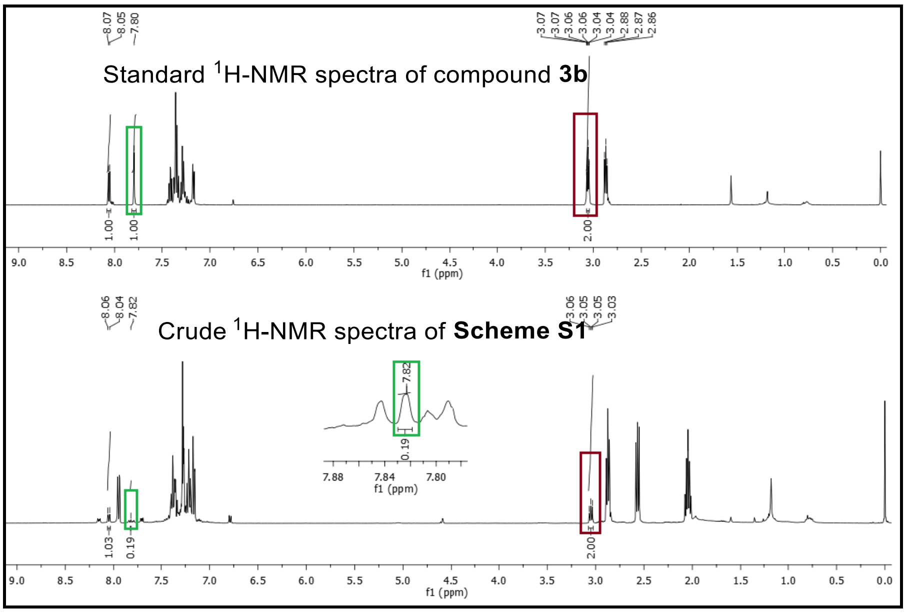

Scheme S2: Determination of rate and order of reaction

Run 1: Reaction was carried out in 1 mL of toluene and yield was calculated by GC

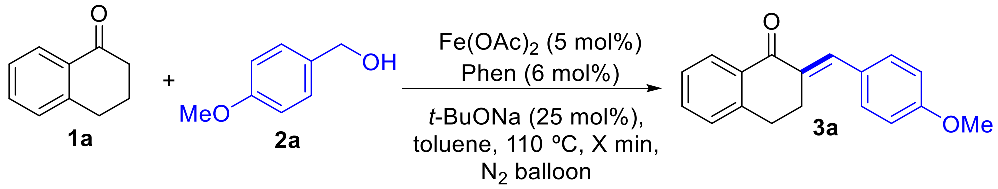

No.	**1a** (mmol)	**2a** (mmol)	$Fe(OAc)_2$ (mmol)	Phen (mmol)	*t*-BuONa (mmol)	Toluene (mL)
Run 1	0.3	0.2	0.01	0.012	0.05	1.0

Sl. No.	Time (min)	Concentration of **2a** (mM)
1	4	190
2	8	179
3	12	172
4	16	165
5	20	160

Run 2: Reaction was carried out in 1 mL of toluene and yield was calculated by GC

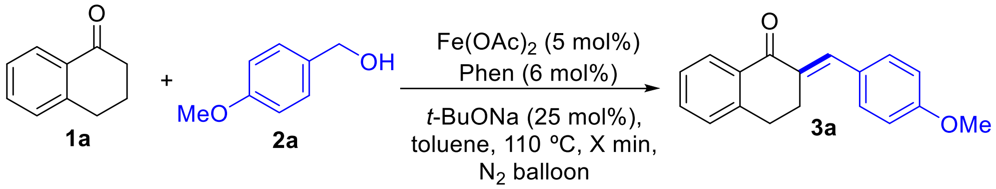

No.	**1a** (mmol)	**2a** (mmol)	$Fe(OAc)_2$ (mmol)	Phen (mmol)	t-BuONa (mmol)	Toluene (mL)
Run 2	0.375	0.25	0.0125	0.015	0.0625	1.0

Sl. No.	Time (min)	Concentration of **2a** (mM)
1	4	229
2	8	216
3	12	203
4	16	196
5	20	191

Considering steady state approximation for benzyl alcohol

From Run 1: $\text{Slope} = k\,[2a]^{x}$

$-1.85 = k\,[0.20]^{x}$

From Run 2: $\text{Slope} = k\,[2a]^{x}$

$-2.4 = k\,[0.25]^{x}$

$-2.4/-1.85 = [0.25]^{x} / [0.2]^{x}$

$1.297 = [1.25]^{x}$

$\text{Log}\,(1.297) = x.\ \text{Log}\,(1.25)$

$x = 0.113 / 0.0969$

$= 1.16 \approx 1$

$\text{Rate} = k\,[2a]^{1}$

Scheme S3: Quantitative determination of hydrogen gas produced in the reaction

In a 10 mL oven dried Schlenk tube, 4-methoxy benzyl alcohol (0.5 mmol), $Fe(OAc)_2$ (5 mol%), Phen (6 mol%), α-tetralone (0.75 mmol) and *t*-BuONa (0.125 mmol), were added followed by toluene 2.0 mL and connected to the gas burette as shown in figure 3. Then the reaction mixture was heated at 110 °C until the production of hydrogen gas ceased. The procedure was repeated three times to get concordant reading.

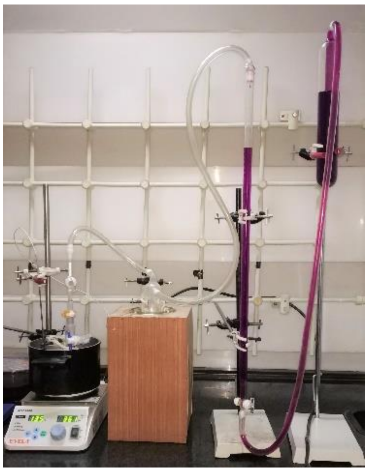

Experimental setup for H_2 determination

Total volume of water displaced, V = 0.007 L

Vapor pressure of water at 298K, P_{H2O} = 23.7695 Torr

Atmospheric pressure at 298K, P_{atm} = 758.3124 Torr

Pressure of H_2 gas, $P_{H2} = P_{atm} - P_{H2O}$ = (758.3124 - 23.7695) Torr

= 734.5429 Torr

$P_{H2} * V = nH_2*R*T$

$nH_2 = P_{H2} * V / R*T$

$= 734.5429 \text{ Torr} * 0.007 \text{ L} / 62.3635 \text{ L Torr K}^{-1} \text{ mol}^{-1} * 298\text{K}$

= 0.000277 mol

= 0.277 mmol

[1.2] Copies of ^{1}H NMR, ^{13}C NMR and HRMS Spectra for selected compounds

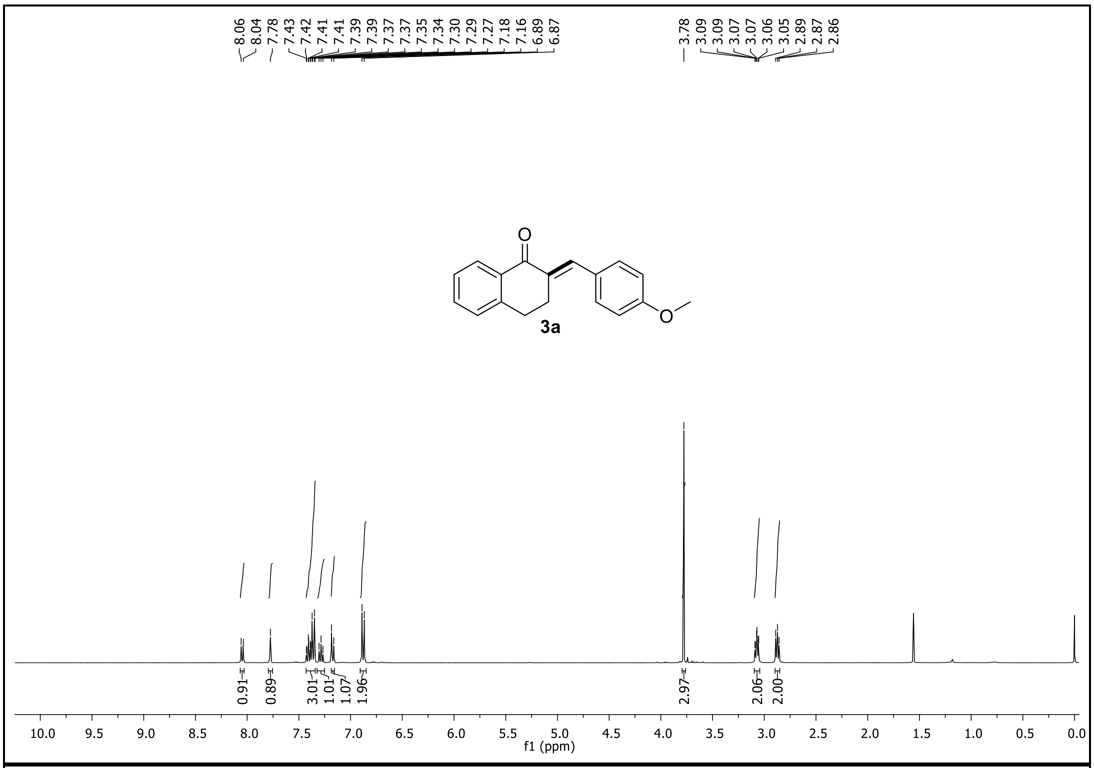

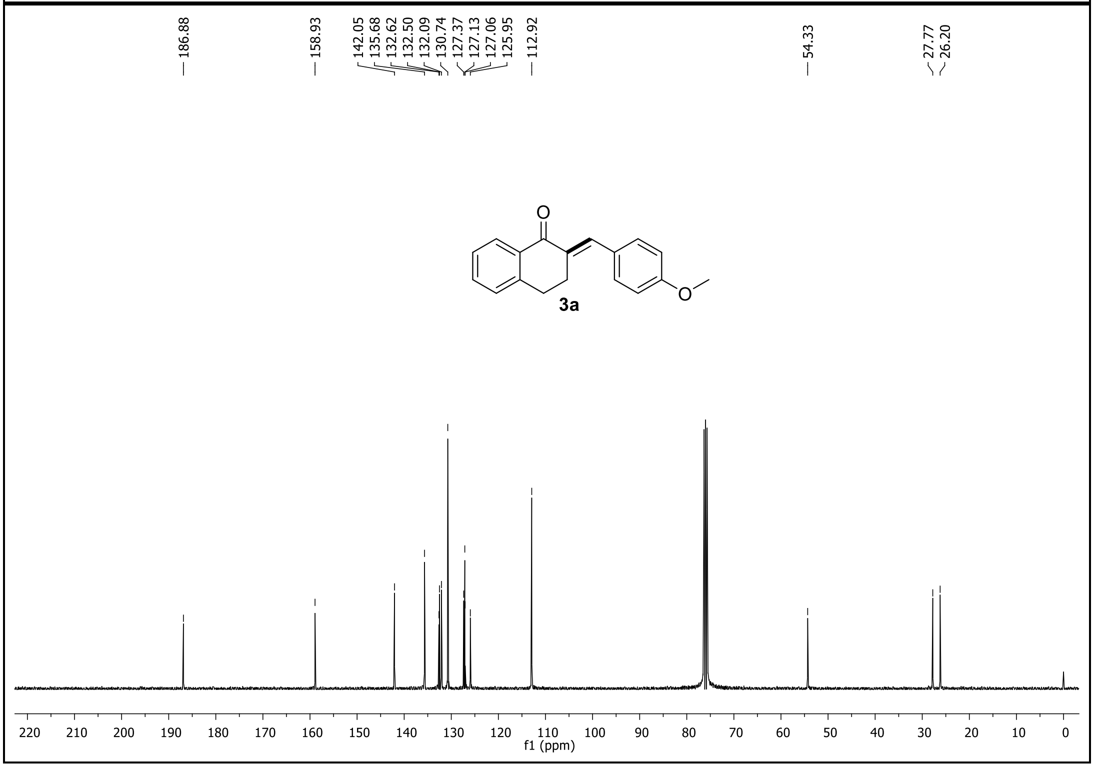

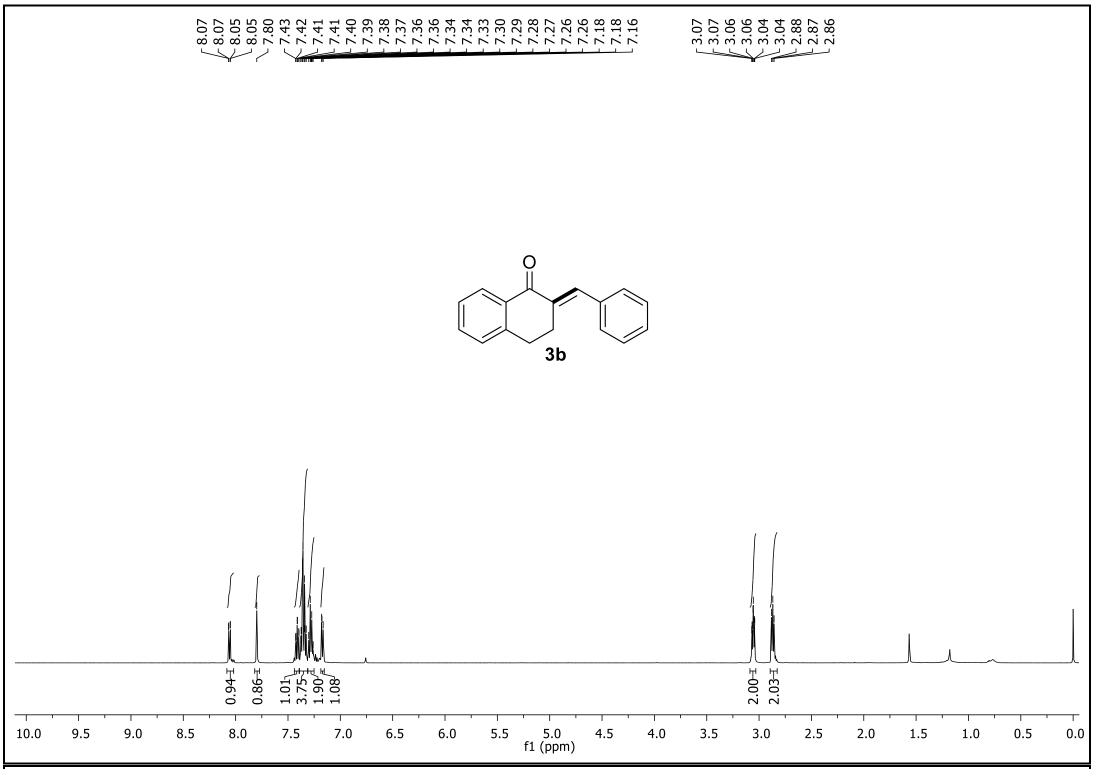
3b
f1 (ppm)

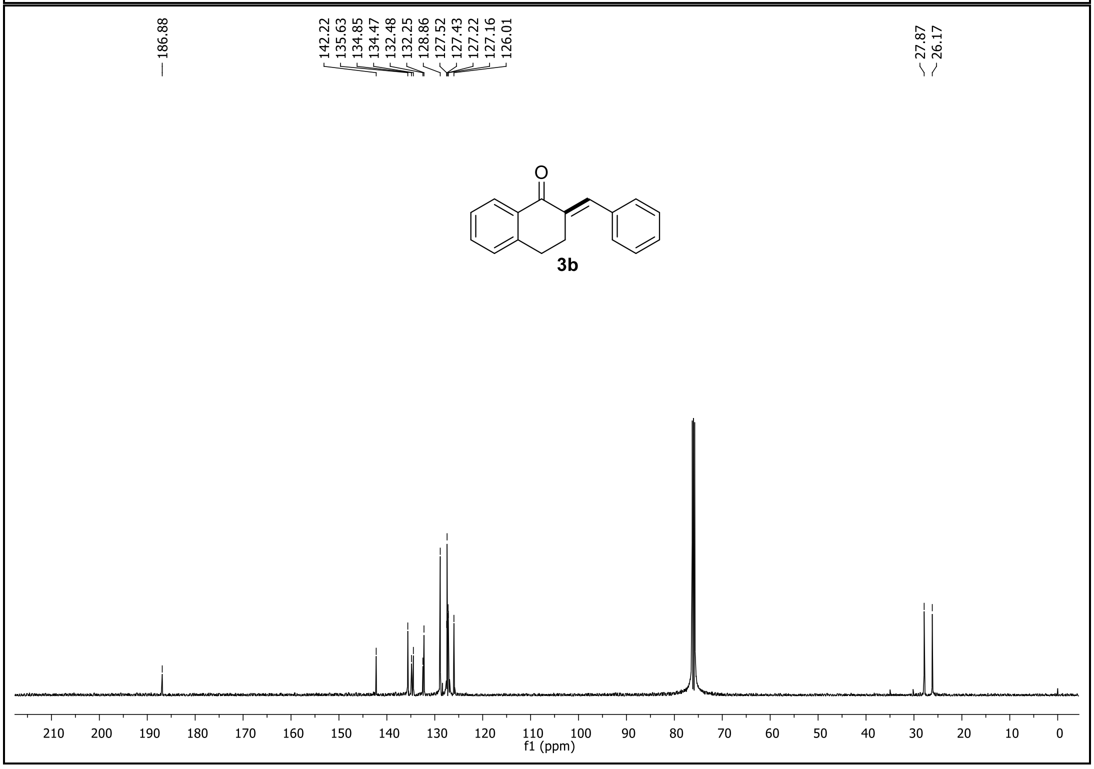
3b
f1 (ppm)

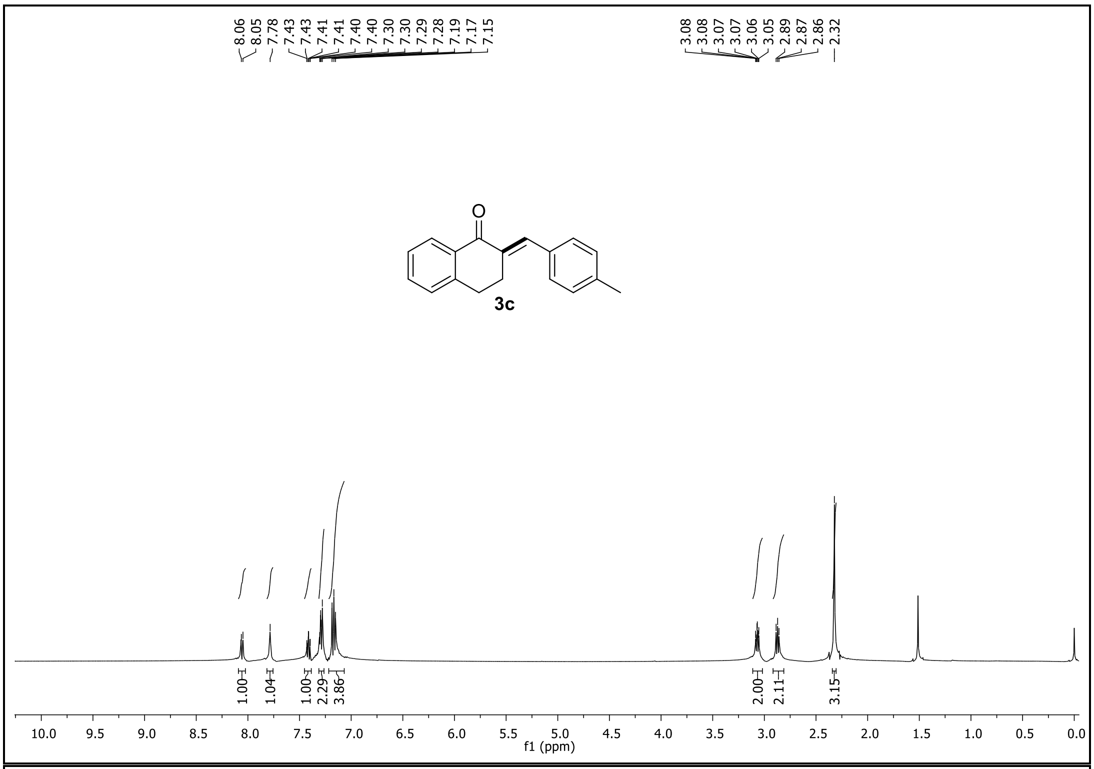
8.06
8.05
7.78
7.43
7.43
7.41
7.41
7.40
7.40
7.30
7.30
7.29
7.28
7.19
7.17
7.15
3.08
3.08
3.07
3.07
3.06
3.05
2.89
2.87
2.86
2.32
O
3c
1.00
1.04
1.00
2.29
3.86
2.00
2.11
3.15
f1 (ppm)

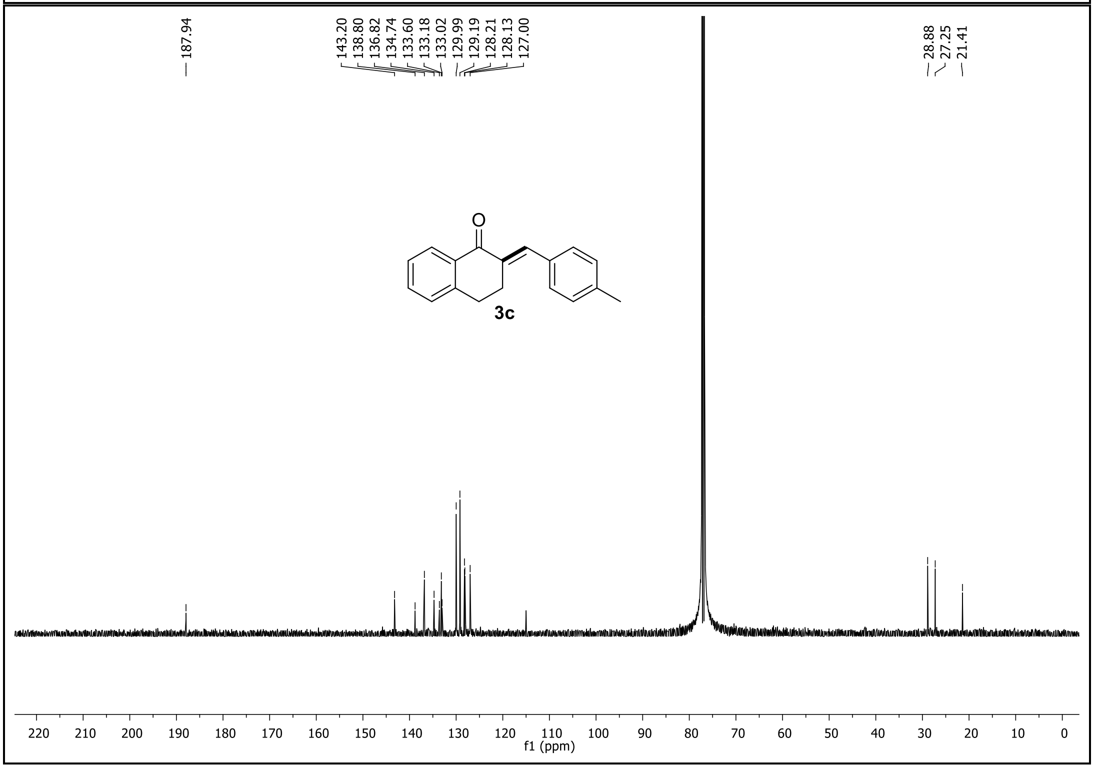
187.94
143.20
138.80
136.82
134.74
133.60
133.18
133.02
129.99
129.19
128.21
128.13
127.00
28.88
27.25
21.41
O
3c
f1 (ppm)

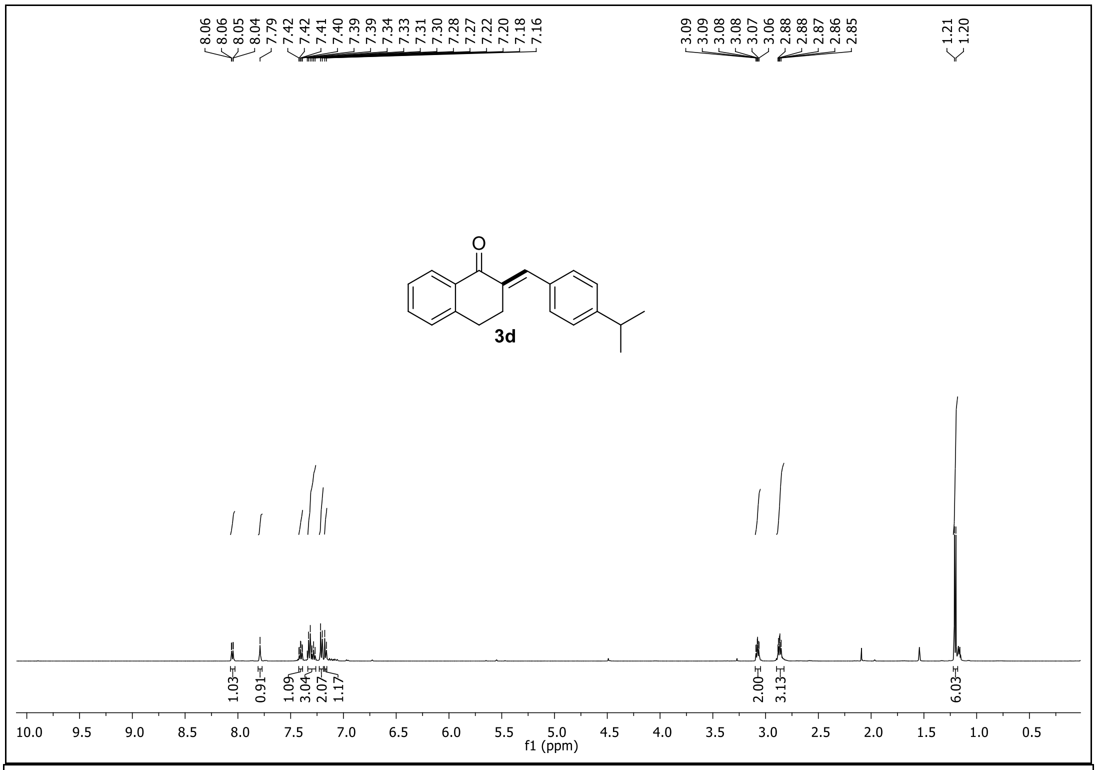
8.06
8.06
8.05
8.04
7.79
7.42
7.42
7.41
7.40
7.39
7.39
7.34
7.33
7.31
7.30
7.28
7.27
7.22
7.20
7.18
7.16
3.09
3.09
3.08
3.08
3.07
3.06
2.88
2.88
2.87
2.86
2.85
1.21
1.20
O
3d
1.03
0.91
1.09
3.04
2.07
1.17
2.00
3.13
6.03
10.0 9.5 9.0 8.5 8.0 7.5 7.0 6.5 6.0 5.5 5.0 4.5 4.0 3.5 3.0 2.5 2.0 1.5 1.0 0.5
f1 (ppm)

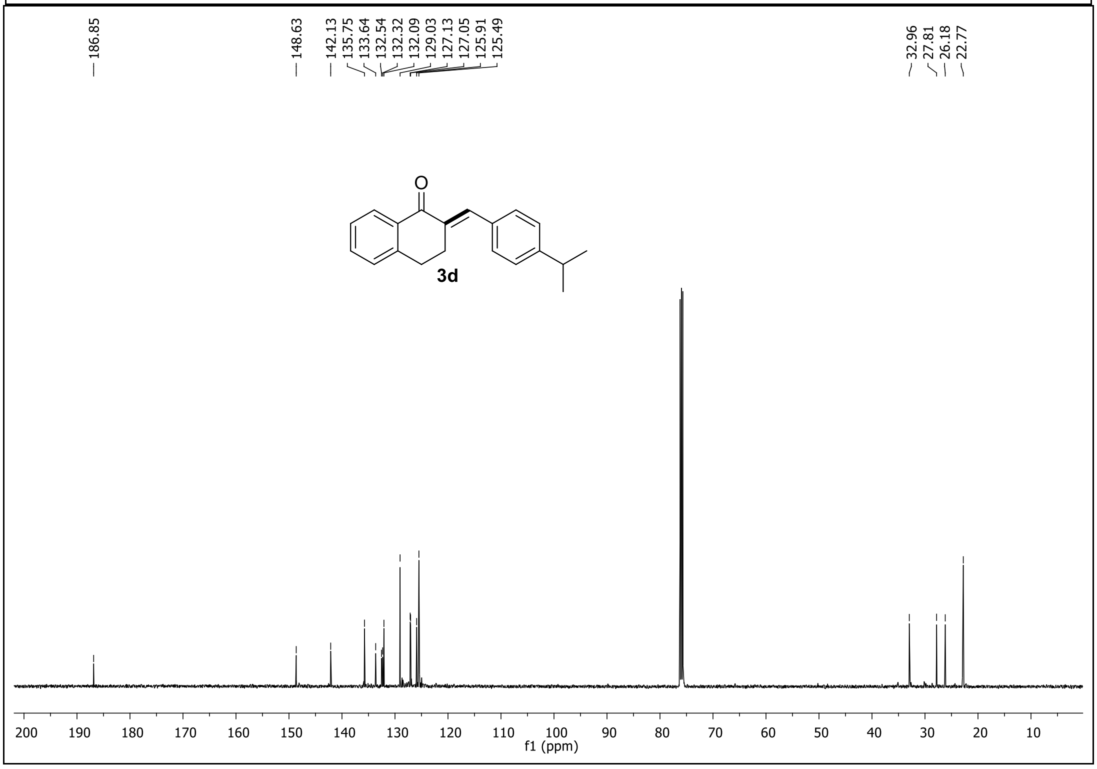
186.85
148.63
142.13
135.75
133.64
132.54
132.32
132.09
129.03
127.13
127.05
125.91
125.49
32.96
27.81
26.18
22.77
O
3d
200 190 180 170 160 150 140 130 120 110 100 90 80 70 60 50 40 30 20 10
f1 (ppm)

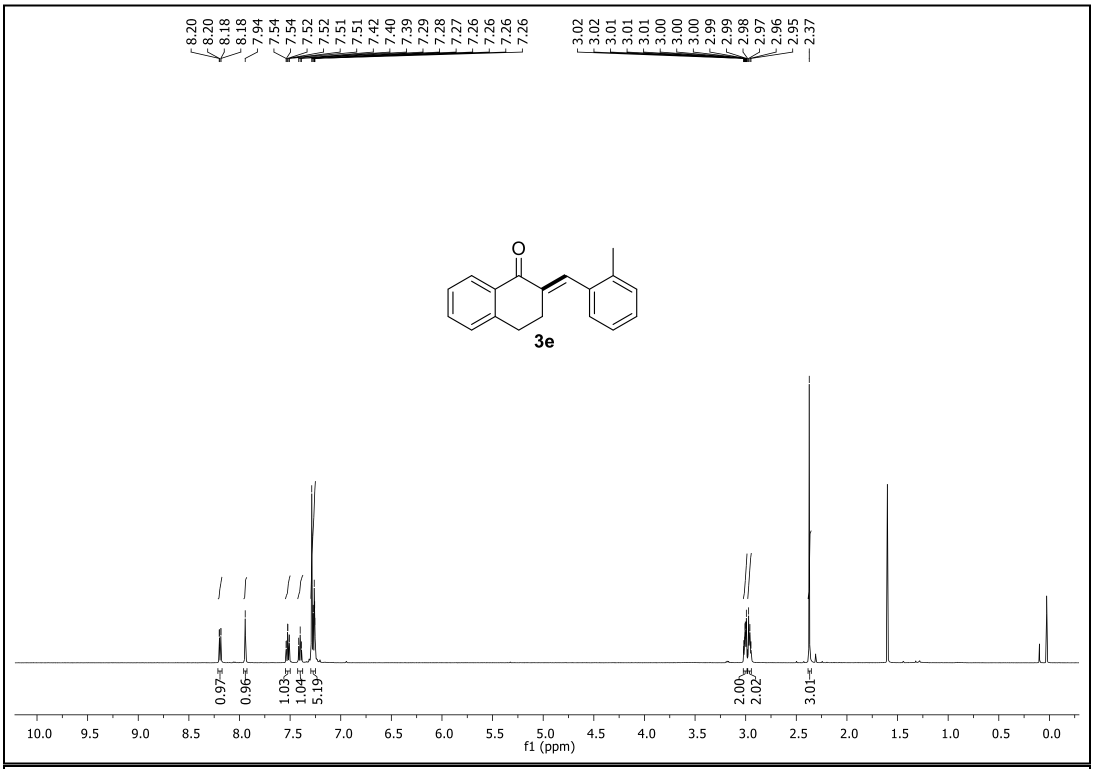
8.20 8.20 8.18 8.18 7.94 7.54 7.54 7.52 7.52 7.51 7.51 7.42 7.40 7.39 7.29 7.28 7.27 7.26 7.26 7.26 7.26
3.02 3.02 3.01 3.01 3.00 3.00 2.99 2.99 2.98 2.97 2.96 2.95 2.37
O
3e
0.97 0.96 1.03 1.04 5.19 2.00 2.02 3.01
10.0 9.5 9.0 8.5 8.0 7.5 7.0 6.5 6.0 5.5 5.0 4.5 4.0 3.5 3.0 2.5 2.0 1.5 1.0 0.5 0.0
f1 (ppm)

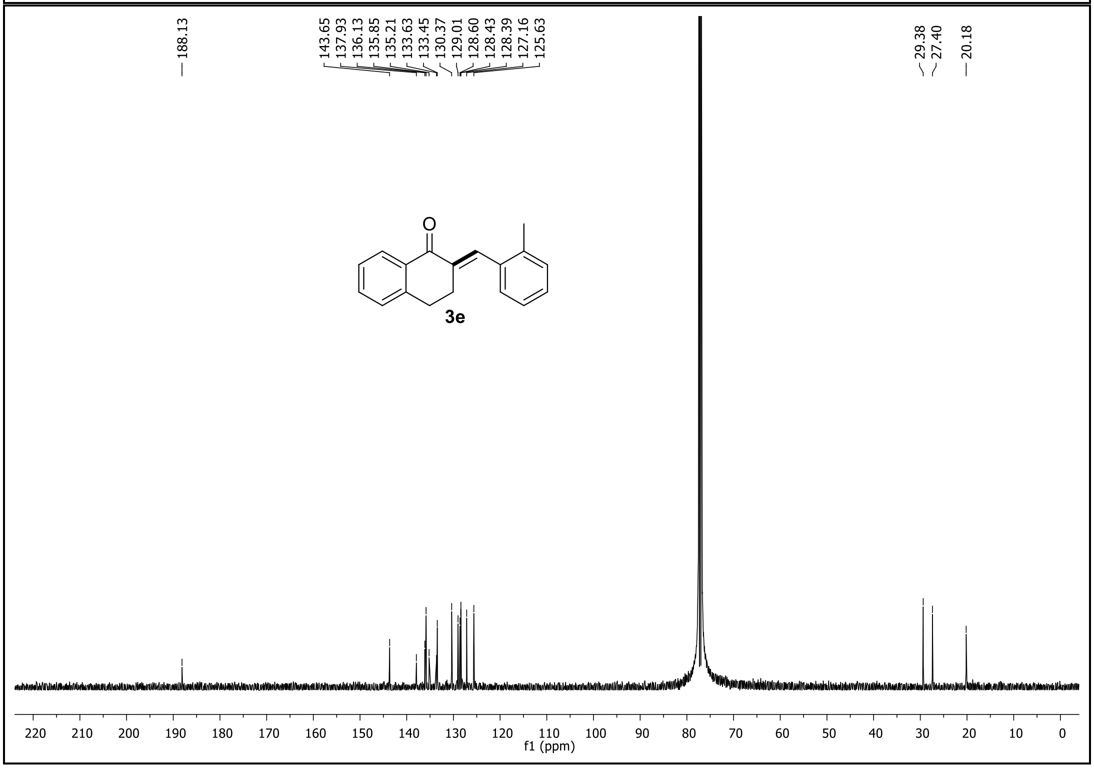
188.13
143.65 137.93 136.13 135.85 135.21 133.63 133.45 130.37 129.01 128.60 128.43 128.39 127.16 125.63
29.38 27.40 20.18
O
3e
220 210 200 190 180 170 160 150 140 130 120 110 100 90 80 70 60 50 40 30 20 10 0
f1 (ppm)

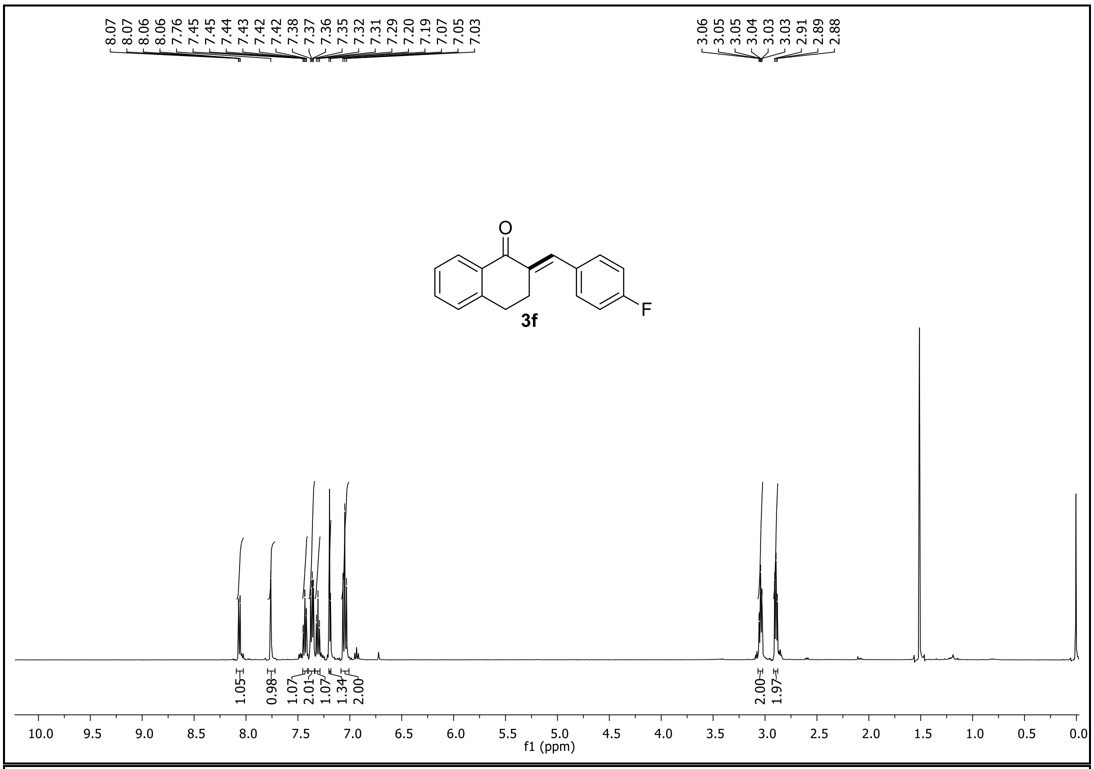
8.07
8.07
8.06
8.06
7.76
7.45
7.45
7.44
7.43
7.42
7.42
7.38
7.37
7.36
7.35
7.32
7.31
7.29
7.20
7.19
7.07
7.05
7.03
3.06
3.05
3.05
3.04
3.03
3.03
2.91
2.89
2.88
O
F
3f
1.05
0.98
1.07
2.01
1.07
1.34
2.00
2.00
1.97
10.0 9.5 9.0 8.5 8.0 7.5 7.0 6.5 6.0 5.5 5.0 4.5 4.0 3.5 3.0 2.5 2.0 1.5 1.0 0.5 0.0
f1 (ppm)
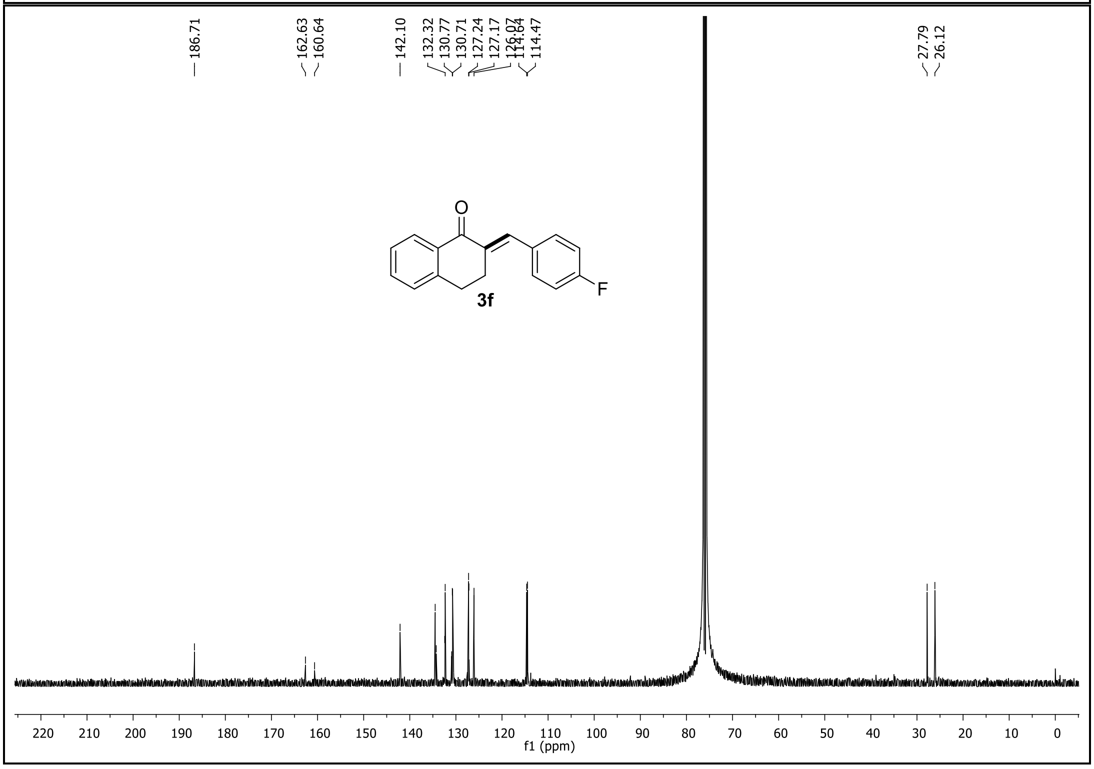
186.71
162.63
160.64
142.10
132.32
130.77
130.71
127.24
127.17
126.07
114.64
114.47
27.79
26.12
O
F
3f
220 210 200 190 180 170 160 150 140 130 120 110 100 90 80 70 60 50 40 30 20 10 0
f1 (ppm)

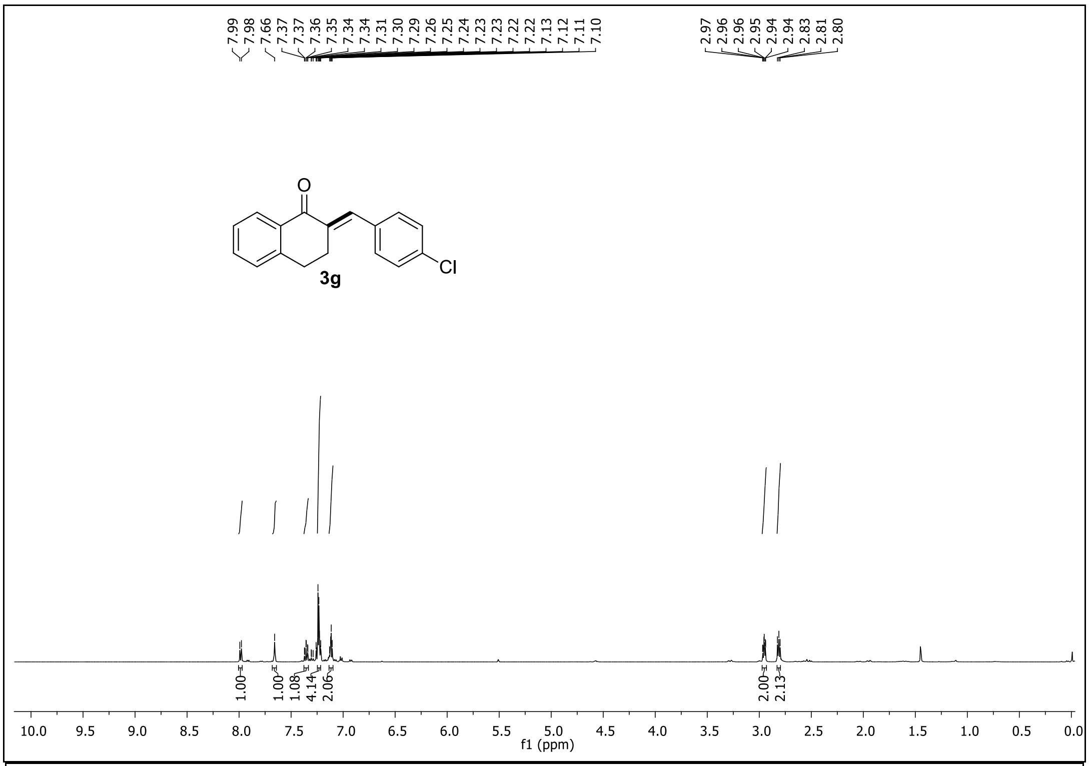
7.99
7.98
7.66
7.37
7.37
7.36
7.35
7.34
7.34
7.31
7.30
7.29
7.26
7.25
7.24
7.23
7.23
7.22
7.22
7.13
7.12
7.11
7.10
2.97
2.96
2.96
2.95
2.94
2.94
2.83
2.81
2.80
O
Cl
3g
1.00
1.00
1.08
4.14
2.06
2.00
2.13
10.0 9.5 9.0 8.5 8.0 7.5 7.0 6.5 6.0 5.5 5.0 4.5 4.0 3.5 3.0 2.5 2.0 1.5 1.0 0.5 0.0
f1 (ppm)

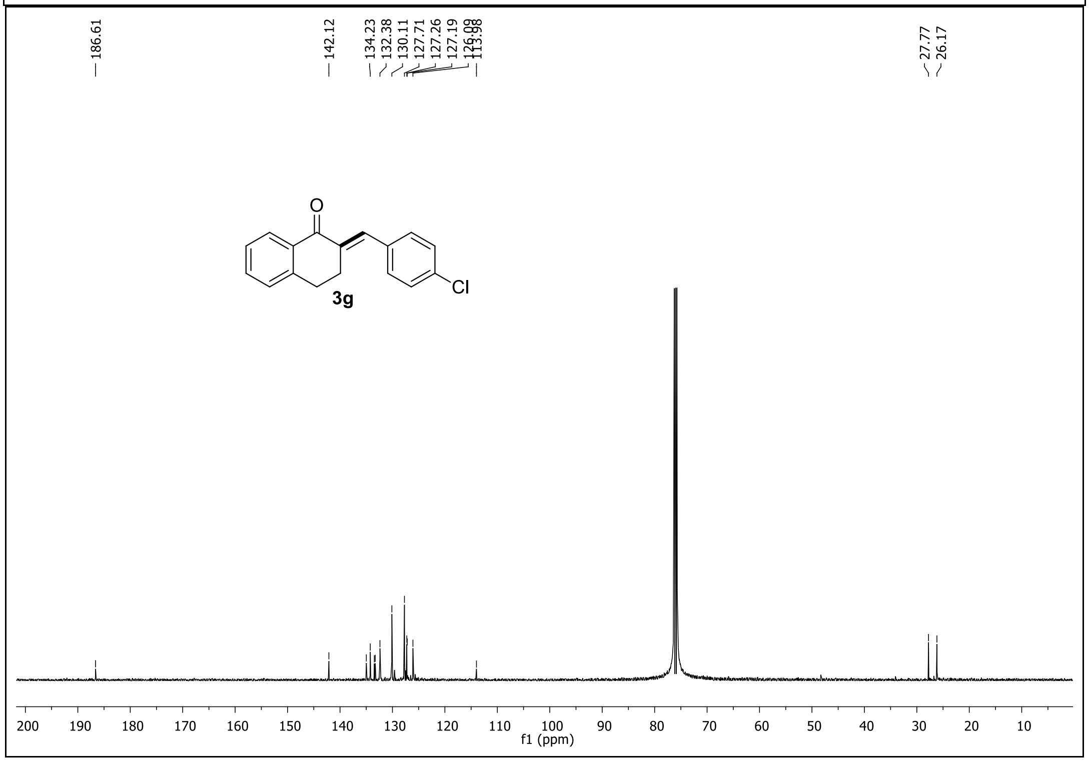
186.61
142.12
134.23
132.38
130.11
127.71
127.26
127.19
126.09
113.98
27.77
26.17
O
Cl
3g
200 190 180 170 160 150 140 130 120 110 100 90 80 70 60 50 40 30 20 10
f1 (ppm)

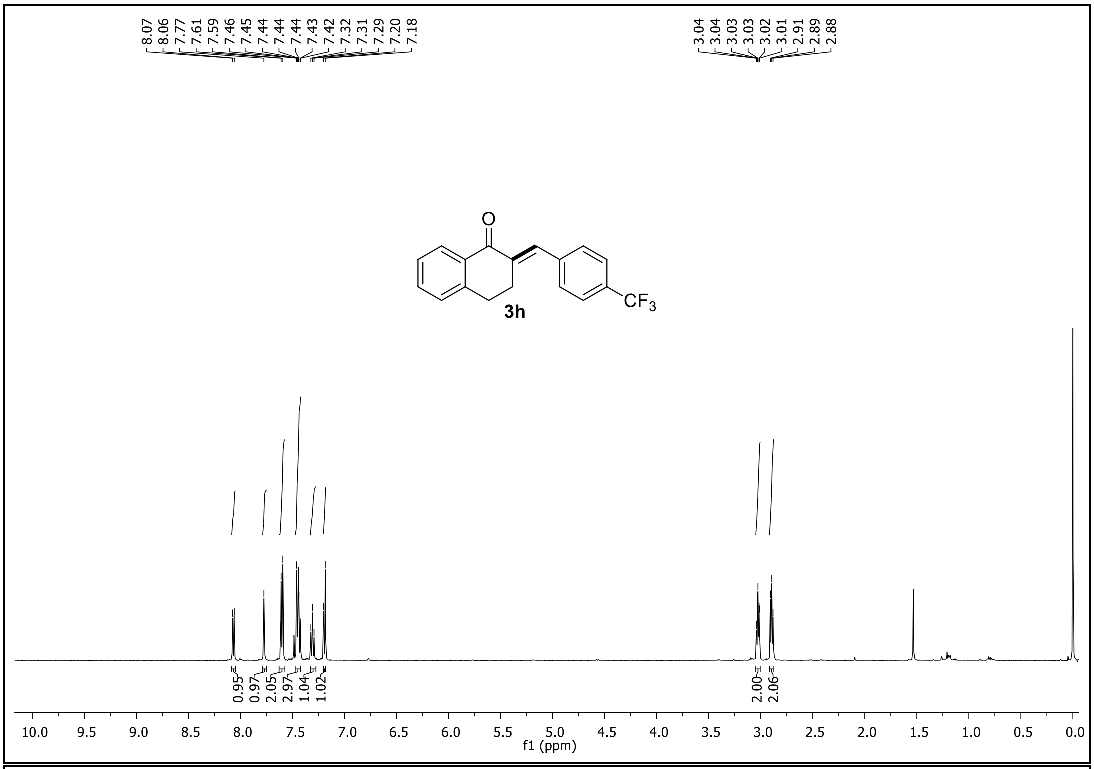
8.07 8.06 7.77 7.61 7.59 7.46 7.45 7.44 7.44 7.44 7.43 7.42 7.32 7.31 7.29 7.20 7.18
3.04 3.04 3.03 3.03 3.02 3.01 2.91 2.89 2.88
O
CF3
3h
0.95 0.97 2.05 2.97 1.04 1.02
2.00 2.06
10.0 9.5 9.0 8.5 8.0 7.5 7.0 6.5 6.0 5.5 5.0 4.5 4.0 3.5 3.0 2.5 2.0 1.5 1.0 0.5 0.0
f1 (ppm)

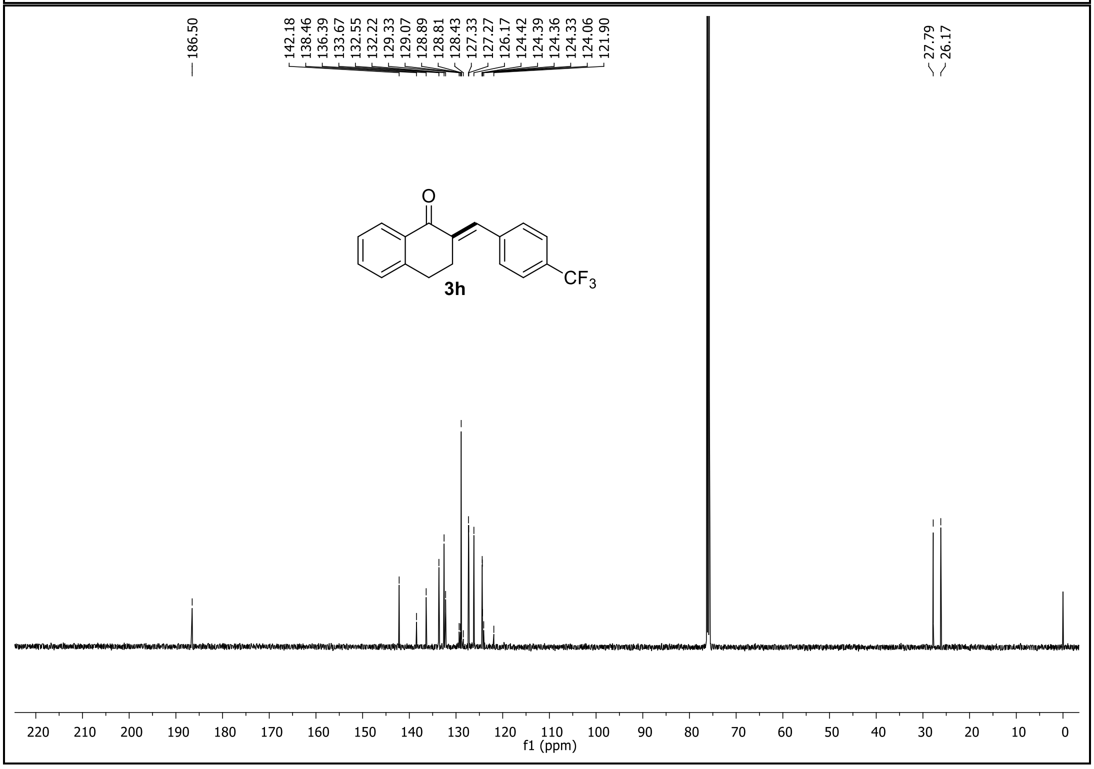
186.50
142.18 138.46 136.39 133.67 132.55 132.22 129.33 129.07 128.89 128.81 128.43 127.33 127.27 126.17 124.42 124.39 124.36 124.33 124.06 121.90
27.79 26.17
O
CF3
3h
220 210 200 190 180 170 160 150 140 130 120 110 100 90 80 70 60 50 40 30 20 10 0
f1 (ppm)

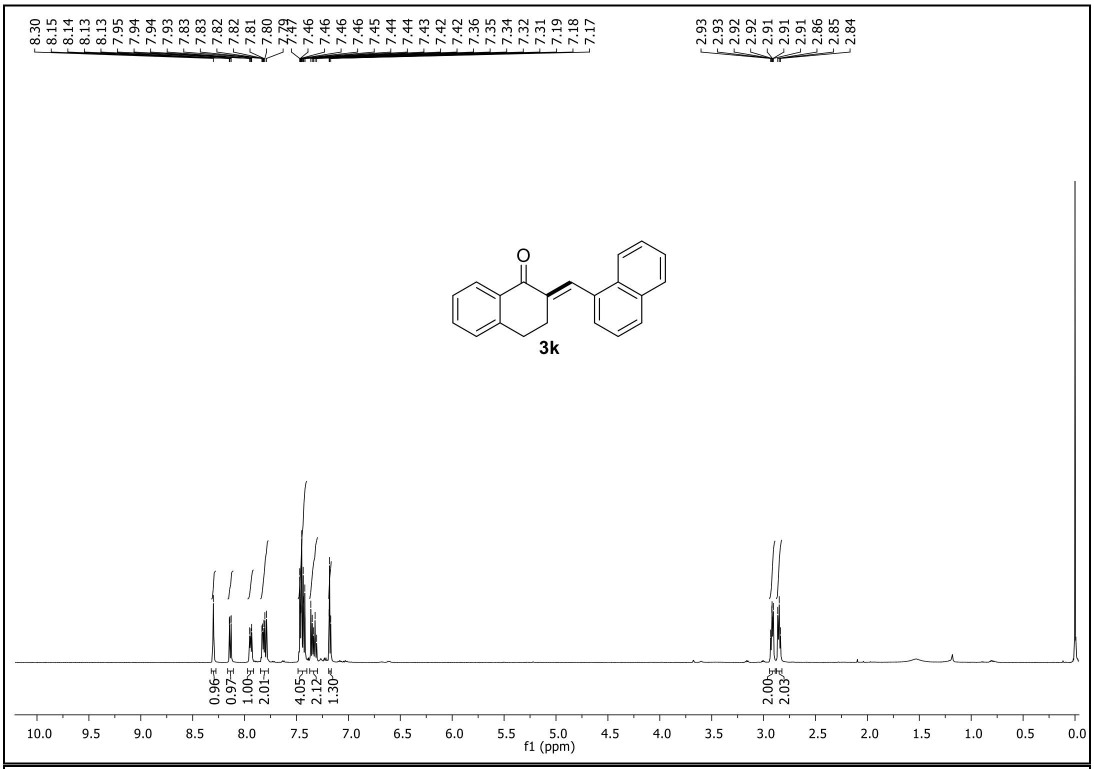
8.30
8.15
8.14
8.13
8.13
7.95
7.94
7.94
7.93
7.83
7.83
7.82
7.82
7.81
7.80
7.79
7.47
7.46
7.46
7.46
7.45
7.44
7.44
7.43
7.42
7.42
7.36
7.35
7.34
7.32
7.31
7.19
7.18
7.17
2.93
2.93
2.92
2.92
2.91
2.91
2.91
2.86
2.85
2.84
O
3k
0.96
0.97
1.00
2.01
4.05
2.12
1.30
2.00
2.03
10.0 9.5 9.0 8.5 8.0 7.5 7.0 6.5 6.0 5.5 5.0 4.5 4.0 3.5 3.0 2.5 2.0 1.5 1.0 0.5 0.0
f1 (ppm)

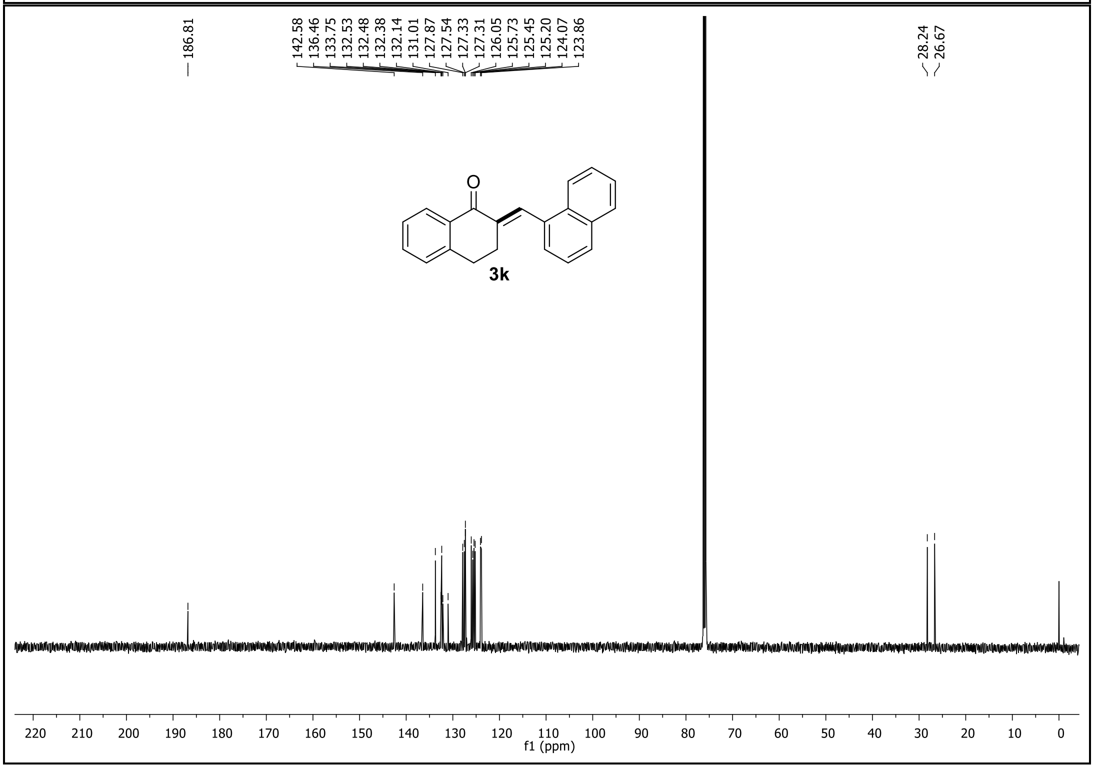
186.81
142.58
136.46
133.75
132.53
132.48
132.38
132.14
131.01
127.87
127.54
127.33
127.31
126.05
125.73
125.45
125.20
124.07
123.86
28.24
26.67
O
3k
220 210 200 190 180 170 160 150 140 130 120 110 100 90 80 70 60 50 40 30 20 10 0
f1 (ppm)

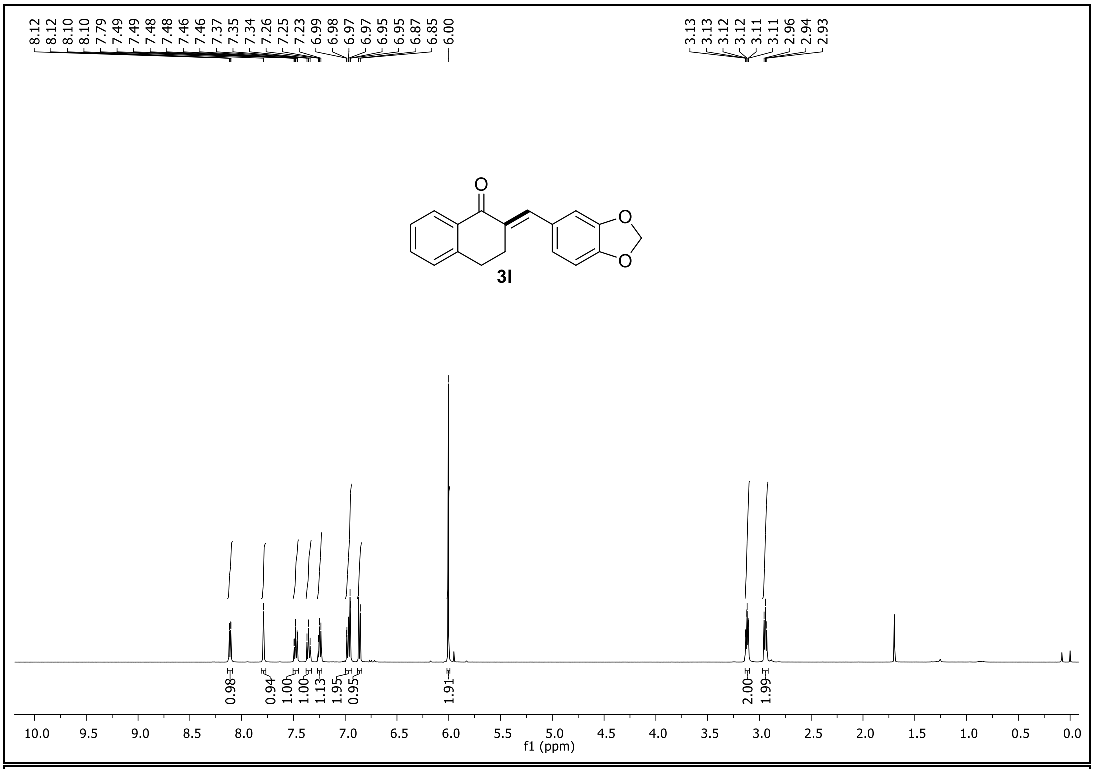
8.12
8.12
8.10
8.10
7.79
7.49
7.48
7.48
7.46
7.46
7.37
7.35
7.34
7.26
7.25
7.23
6.99
6.98
6.97
6.97
6.95
6.95
6.87
6.85
6.00
3.13
3.13
3.12
3.12
3.11
3.11
2.96
2.94
2.93
O
O
O
3l
0.98
0.94
1.00
1.00
1.13
1.95
0.95
1.91
2.00
1.99
10.0
9.5
9.0
8.5
8.0
7.5
7.0
6.5
6.0
5.5
5.0
4.5
4.0
3.5
3.0
2.5
2.0
1.5
1.0
0.5
0.0
f1 (ppm)

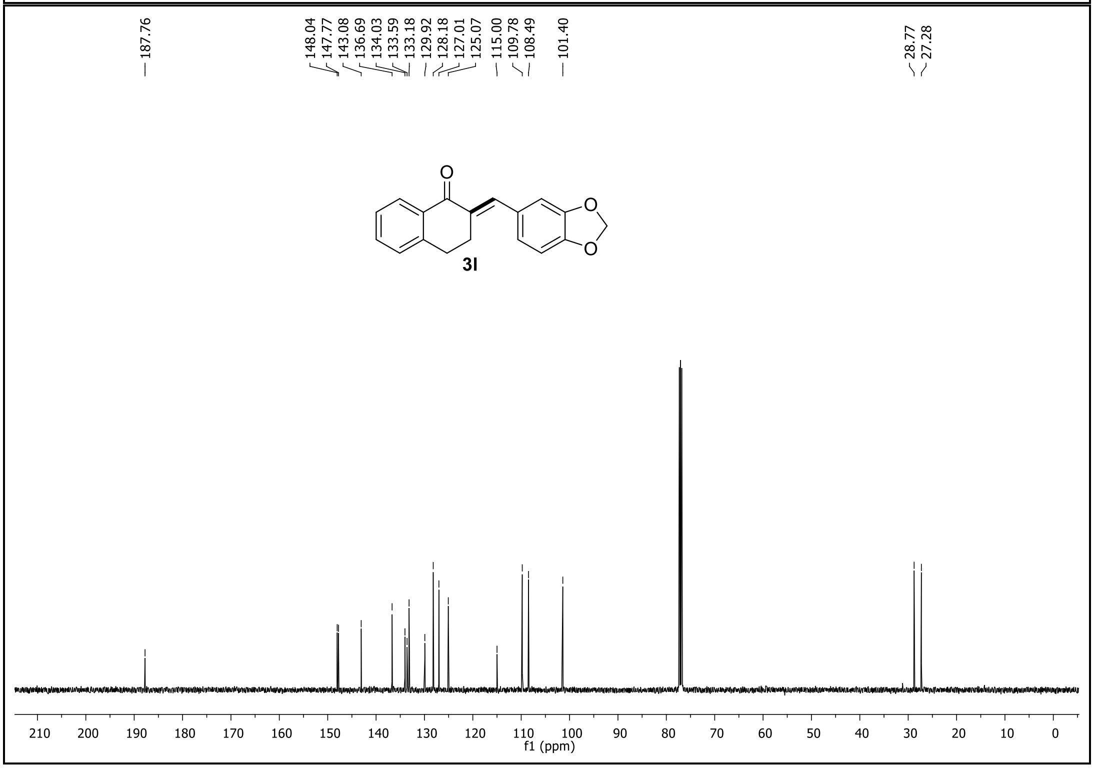
187.76
148.04
147.77
143.08
136.69
134.03
133.59
133.18
129.92
128.18
127.01
125.07
115.00
109.78
108.49
101.40
28.77
27.28
O
O
O
3l
210
200
190
180
170
160
150
140
130
120
110
100
90
80
70
60
50
40
30
20
10
0
f1 (ppm)

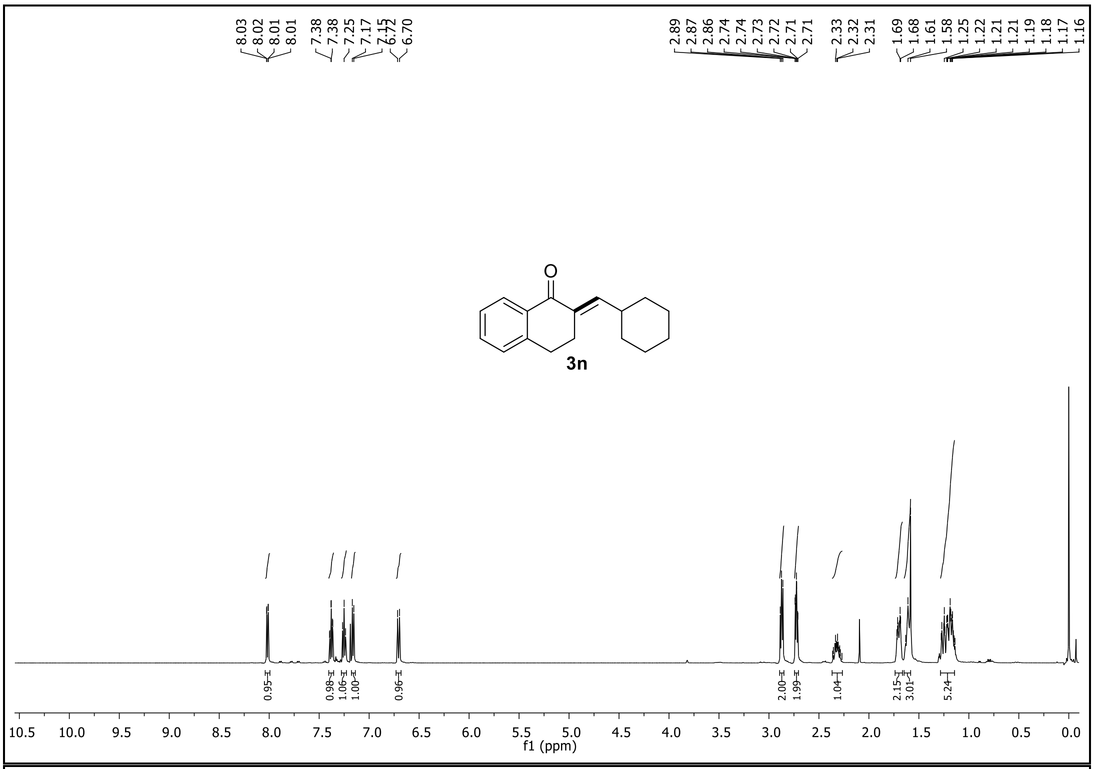

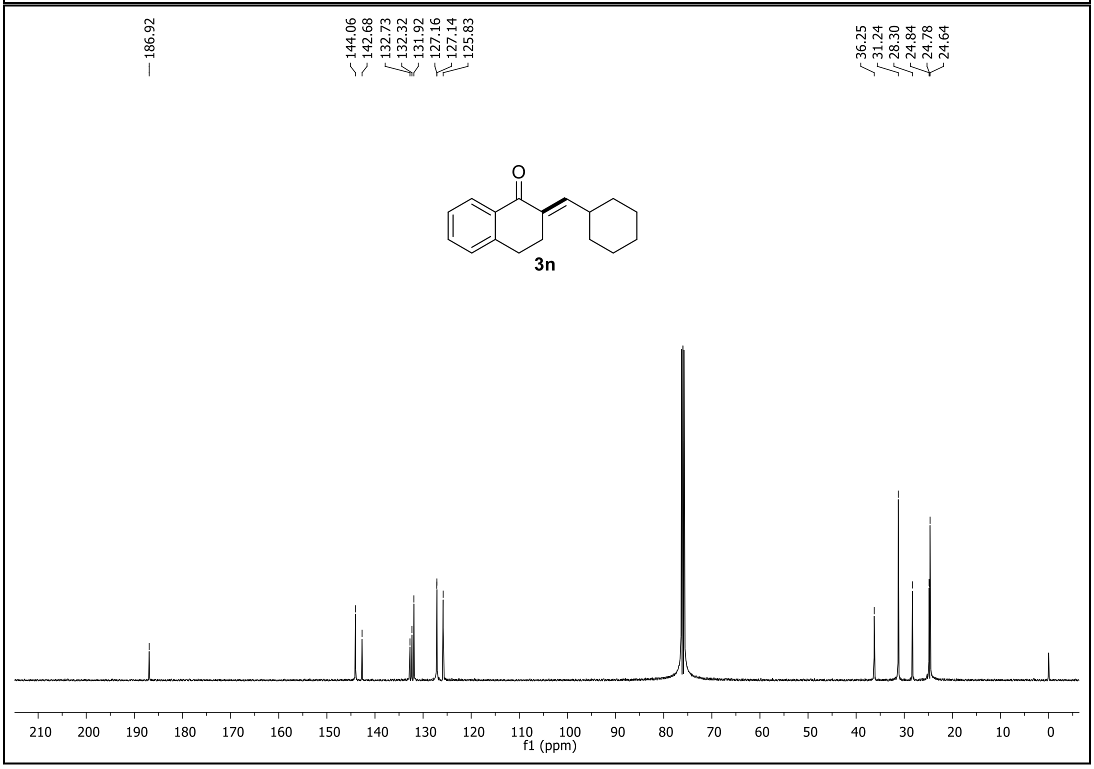

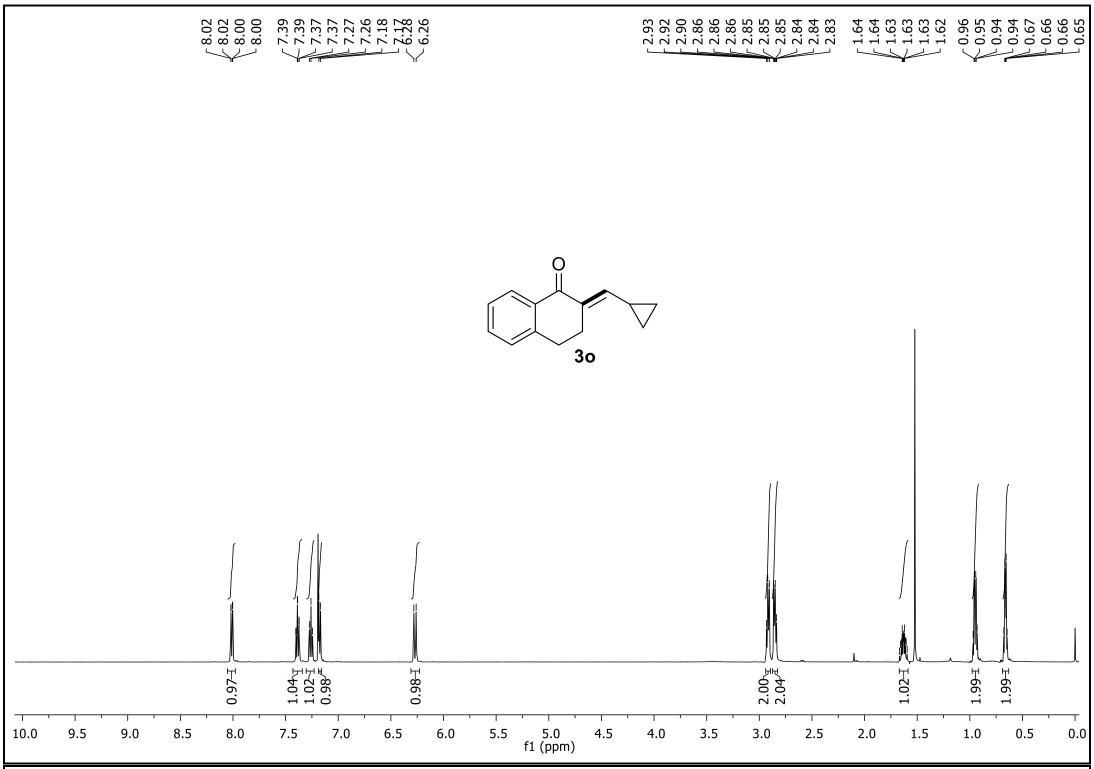
8.02
8.02
8.00
8.00
7.39
7.39
7.37
7.37
7.27
7.26
7.18
7.17
6.28
6.26
2.93
2.92
2.90
2.86
2.86
2.86
2.85
2.85
2.85
2.84
2.84
2.83
1.64
1.64
1.63
1.63
1.63
1.62
0.96
0.95
0.94
0.94
0.67
0.66
0.66
0.65
O
3o
0.97
1.04
1.02
0.98
0.98
2.00
2.04
1.02
1.99
1.99
10.0 9.5 9.0 8.5 8.0 7.5 7.0 6.5 6.0 5.5 5.0 4.5 4.0 3.5 3.0 2.5 2.0 1.5 1.0 0.5 0.0
f1 (ppm)

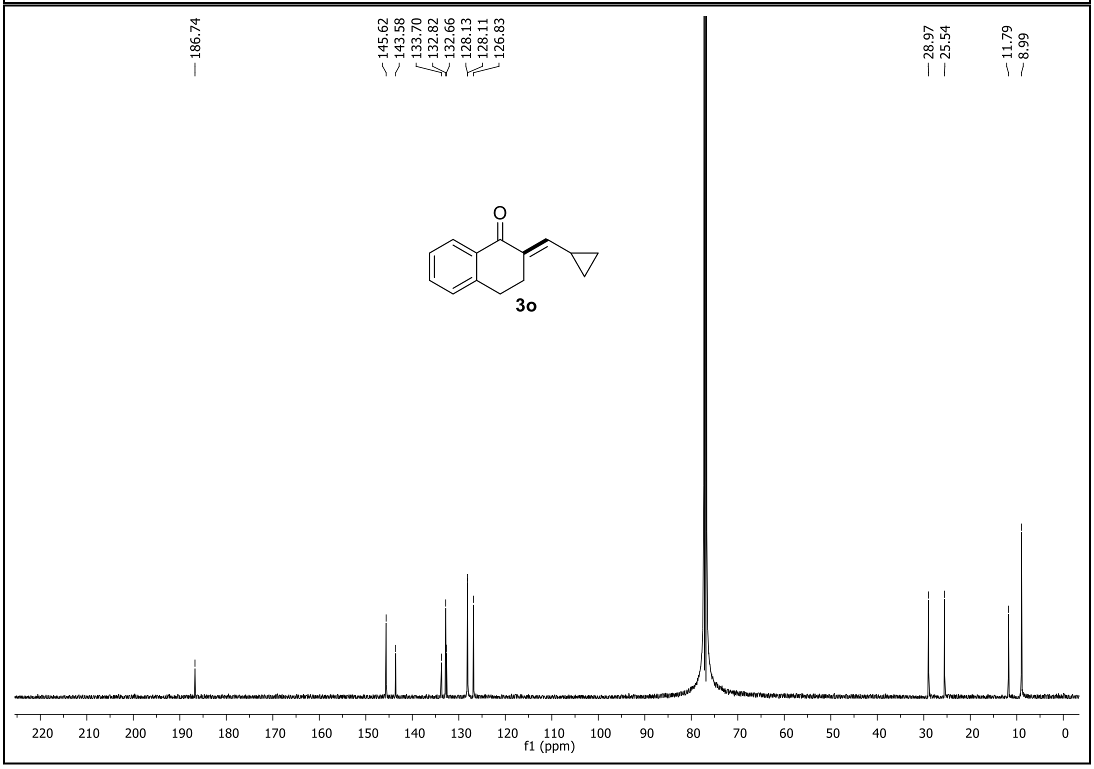
186.74
145.62
143.58
133.70
132.82
132.66
128.13
128.11
126.83
28.97
25.54
11.79
8.99
O
3o
220 210 200 190 180 170 160 150 140 130 120 110 100 90 80 70 60 50 40 30 20 10 0
f1 (ppm)

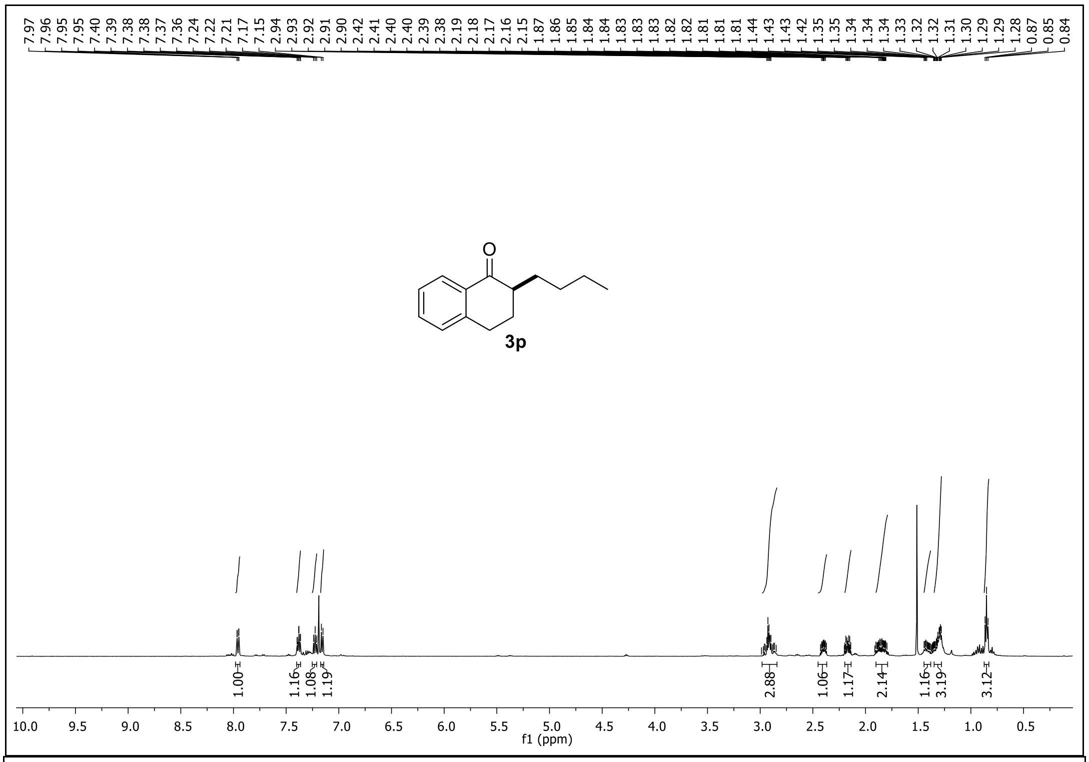
7.97 7.96 7.95 7.95 7.40 7.39 7.38 7.38 7.37 7.36 7.24 7.22 7.21 7.17 7.15 2.94 2.93 2.92 2.91 2.90 2.42 2.41 2.40 2.40 2.39 2.38 2.19 2.18 2.17 2.16 2.15 1.87 1.86 1.85 1.84 1.84 1.83 1.83 1.83 1.82 1.82 1.81 1.81 1.81 1.44 1.43 1.43 1.42 1.35 1.35 1.34 1.34 1.34 1.33 1.32 1.32 1.31 1.30 1.29 1.29 1.28 0.87 0.85 0.84
O
3p
1.00 1.16 1.08 1.19 2.88 1.06 1.17 2.14 1.16 3.19 3.12
10.0 9.5 9.0 8.5 8.0 7.5 7.0 6.5 6.0 5.5 5.0 4.5 4.0 3.5 3.0 2.5 2.0 1.5 1.0 0.5
f1 (ppm)

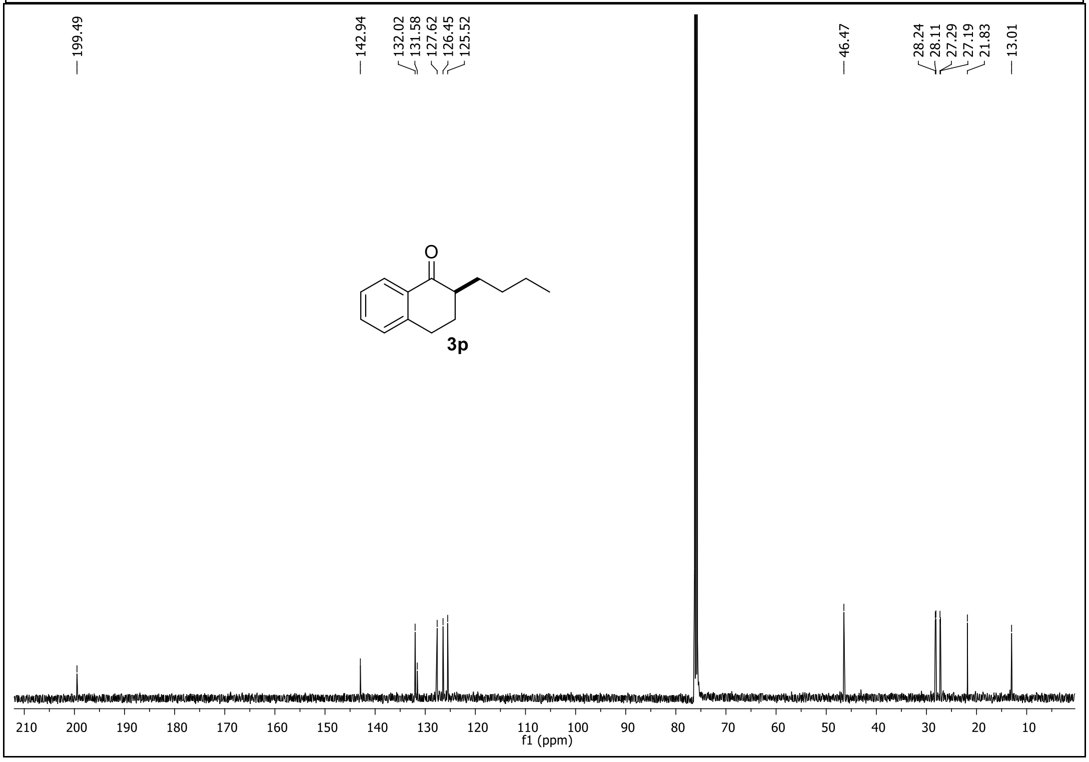
199.49
142.94
132.02 131.58 127.62 126.45 125.52
46.47
28.24 28.11 27.29 27.19 21.83
13.01
O
3p
210 200 190 180 170 160 150 140 130 120 110 100 90 80 70 60 50 40 30 20 10
f1 (ppm)

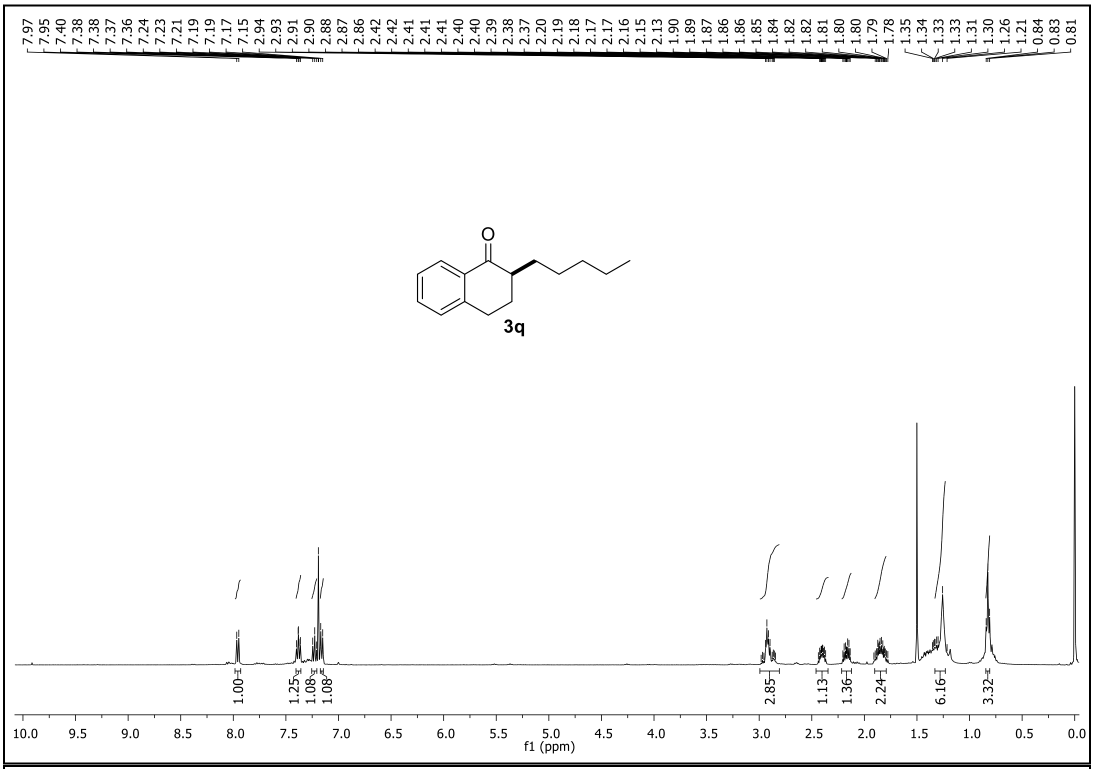
7.97 7.95 7.40 7.38 7.38 7.37 7.36 7.24 7.23 7.21 7.19 7.19 7.17 7.15 2.94 2.93 2.91 2.90 2.88 2.87 2.86 2.42 2.42 2.41 2.41 2.40 2.40 2.39 2.38 2.37 2.20 2.19 2.18 2.17 2.17 2.16 2.15 2.13 1.90 1.89 1.87 1.86 1.85 1.84 1.82 1.82 1.81 1.80 1.80 1.79 1.78 1.35 1.34 1.33 1.33 1.31 1.30 1.26 1.21 0.84 0.83 0.81
O
3q
1.00 1.25 1.08 1.08 2.85 1.13 1.36 2.24 6.16 3.32
10.0 9.5 9.0 8.5 8.0 7.5 7.0 6.5 6.0 5.5 5.0 4.5 4.0 3.5 3.0 2.5 2.0 1.5 1.0 0.5 0.0
f1 (ppm)

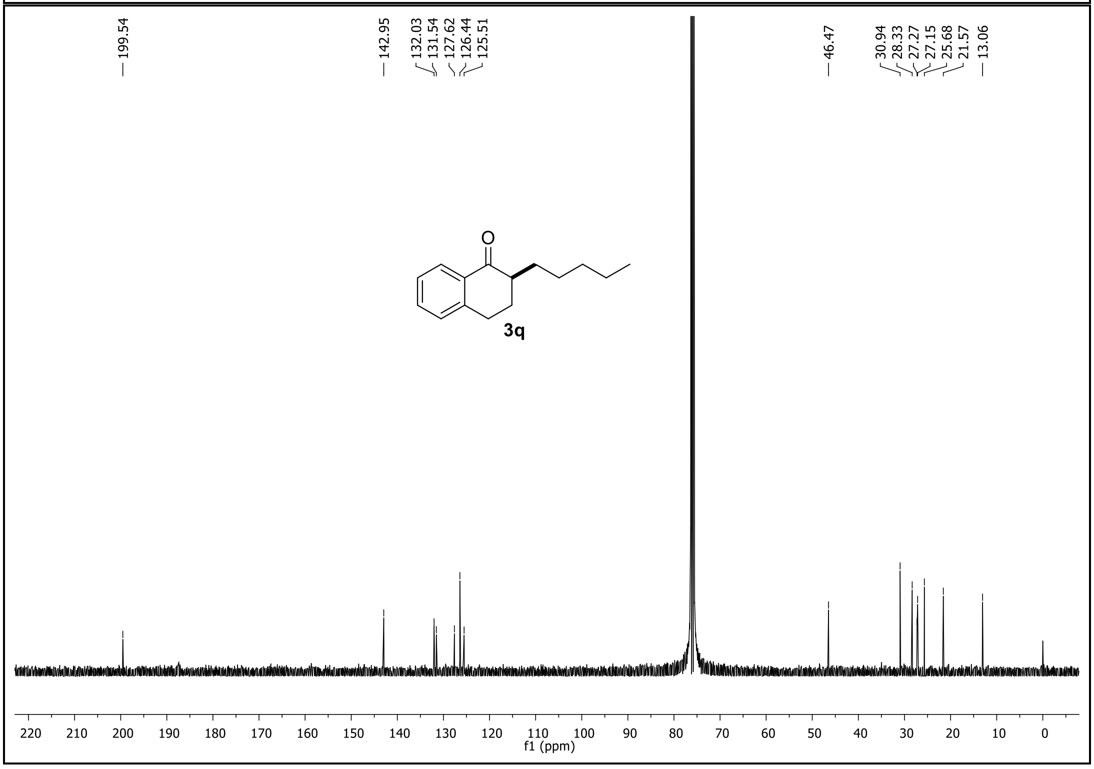
199.54 142.95 132.03 131.54 127.62 126.44 125.51 46.47 30.94 28.33 27.27 27.15 25.68 21.57 13.06
O
3q
220 210 200 190 180 170 160 150 140 130 120 110 100 90 80 70 60 50 40 30 20 10 0
f1 (ppm)

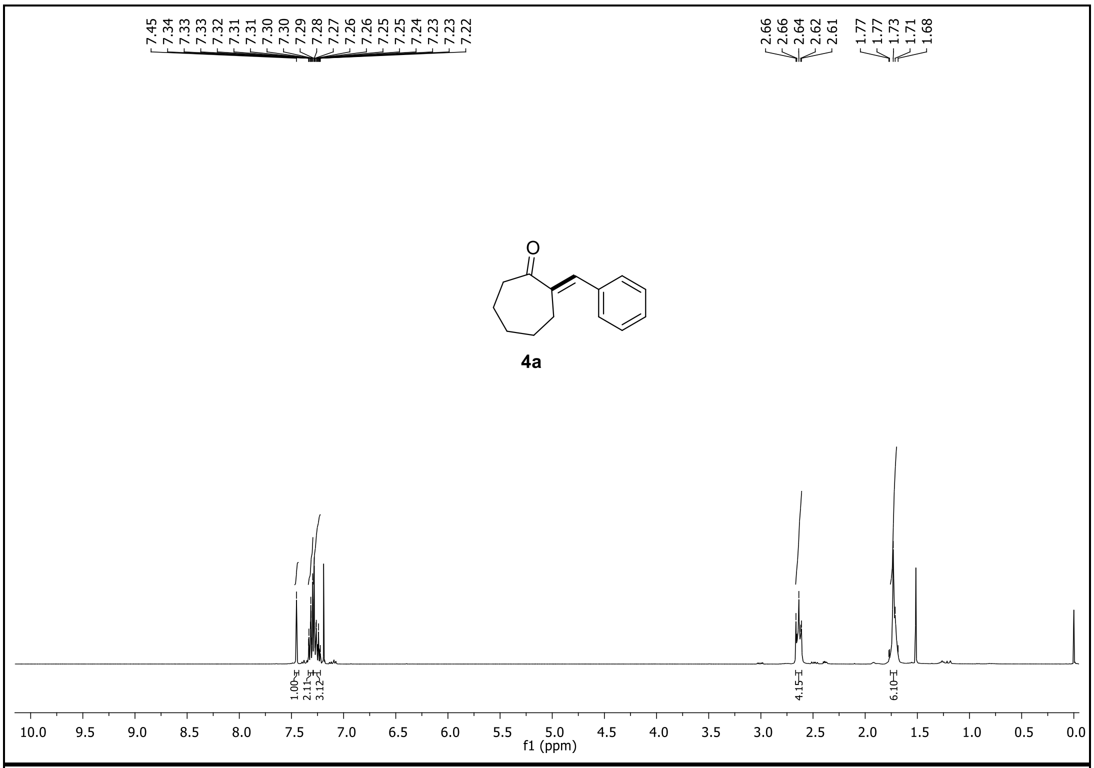
7.45 7.34 7.33 7.33 7.32 7.31 7.31 7.30 7.29 7.28 7.27 7.26 7.26 7.25 7.25 7.24 7.23 7.23 7.22
2.66 2.66 2.64 2.62 2.61 1.77 1.77 1.73 1.71 1.68
O
4a
1.00 2.11 3.12 4.15 6.10
10.0 9.5 9.0 8.5 8.0 7.5 7.0 6.5 6.0 5.5 5.0 4.5 4.0 3.5 3.0 2.5 2.0 1.5 1.0 0.5 0.0
f1 (ppm)

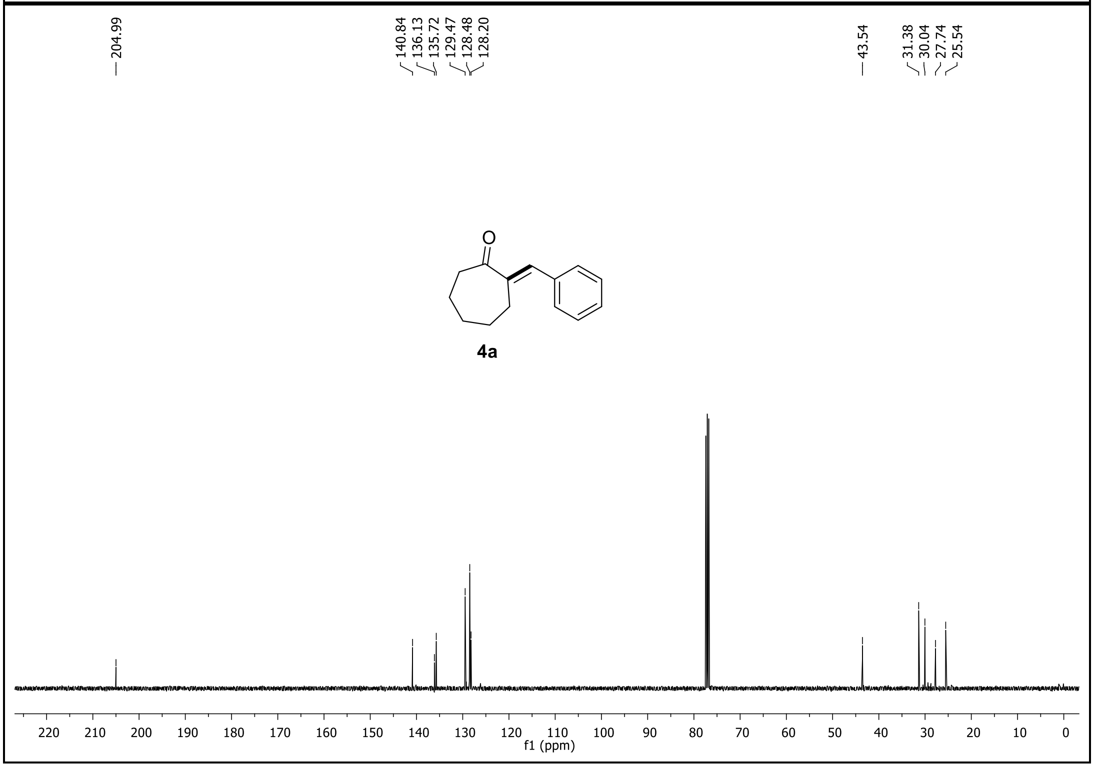
204.99
140.84 136.13 135.72 129.47 128.48 128.20
43.54
31.38 30.04 27.74 25.54
O
4a
220 210 200 190 180 170 160 150 140 130 120 110 100 90 80 70 60 50 40 30 20 10 0
f1 (ppm)

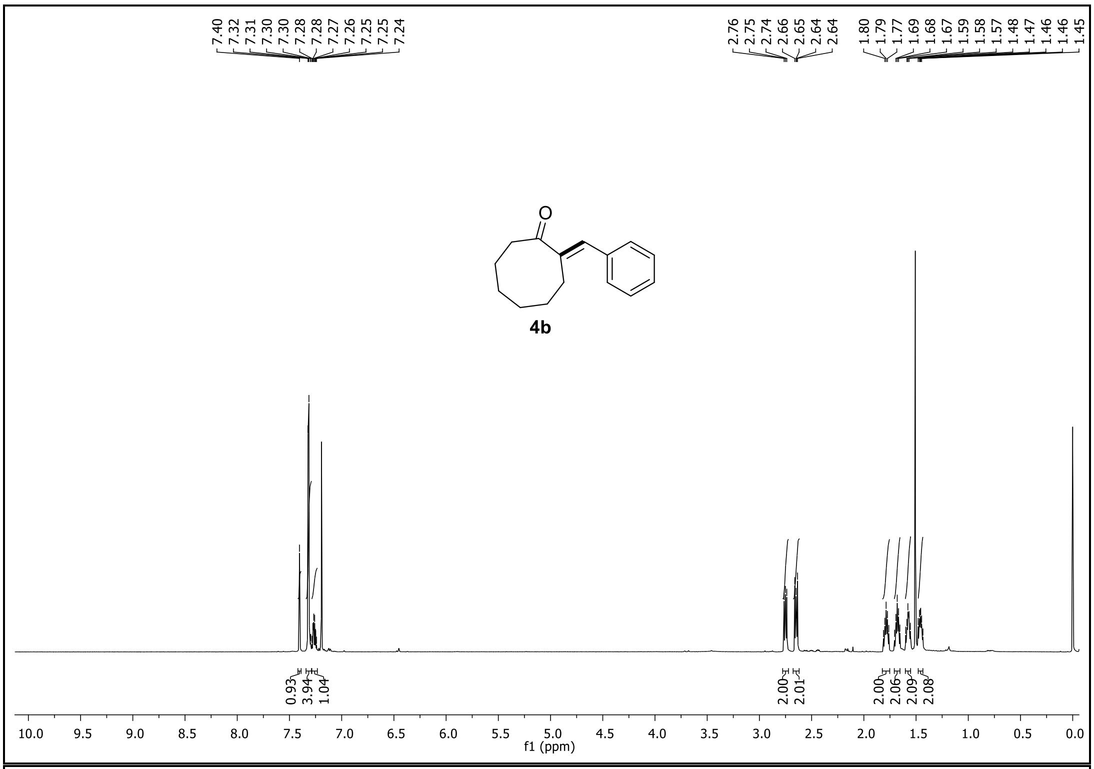
7.40 7.32 7.31 7.30 7.30 7.28 7.28 7.27 7.26 7.25 7.25 7.24
2.76 2.75 2.74 2.66 2.65 2.64 2.64
1.80 1.79 1.77 1.69 1.68 1.67 1.59 1.58 1.57 1.48 1.47 1.46 1.46 1.45
O
4b
0.93 3.94 1.04
2.00 2.01
2.00 2.06 2.09 2.08
10.0 9.5 9.0 8.5 8.0 7.5 7.0 6.5 6.0 5.5 5.0 4.5 4.0 3.5 3.0 2.5 2.0 1.5 1.0 0.5 0.0
f1 (ppm)

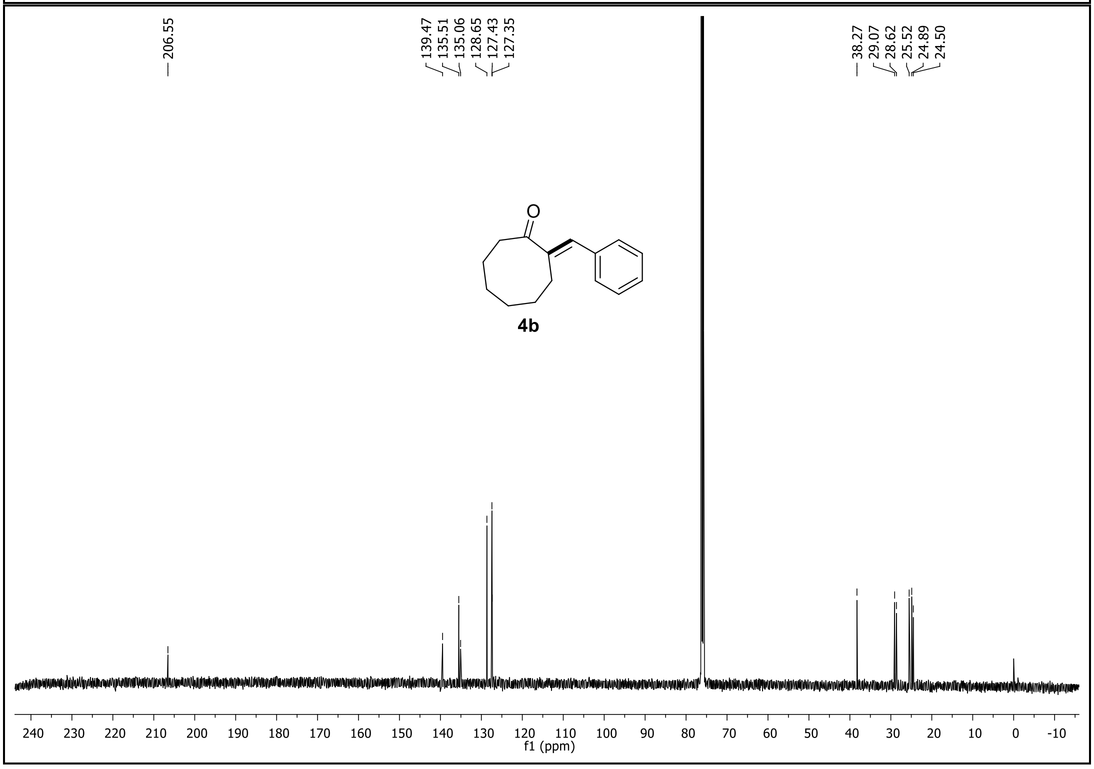
206.55
139.47 135.51 135.06 128.65 127.43 127.35
38.27 29.07 28.62 25.52 24.89 24.50
O
4b
240 230 220 210 200 190 180 170 160 150 140 130 120 110 100 90 80 70 60 50 40 30 20 10 0 -10
f1 (ppm)

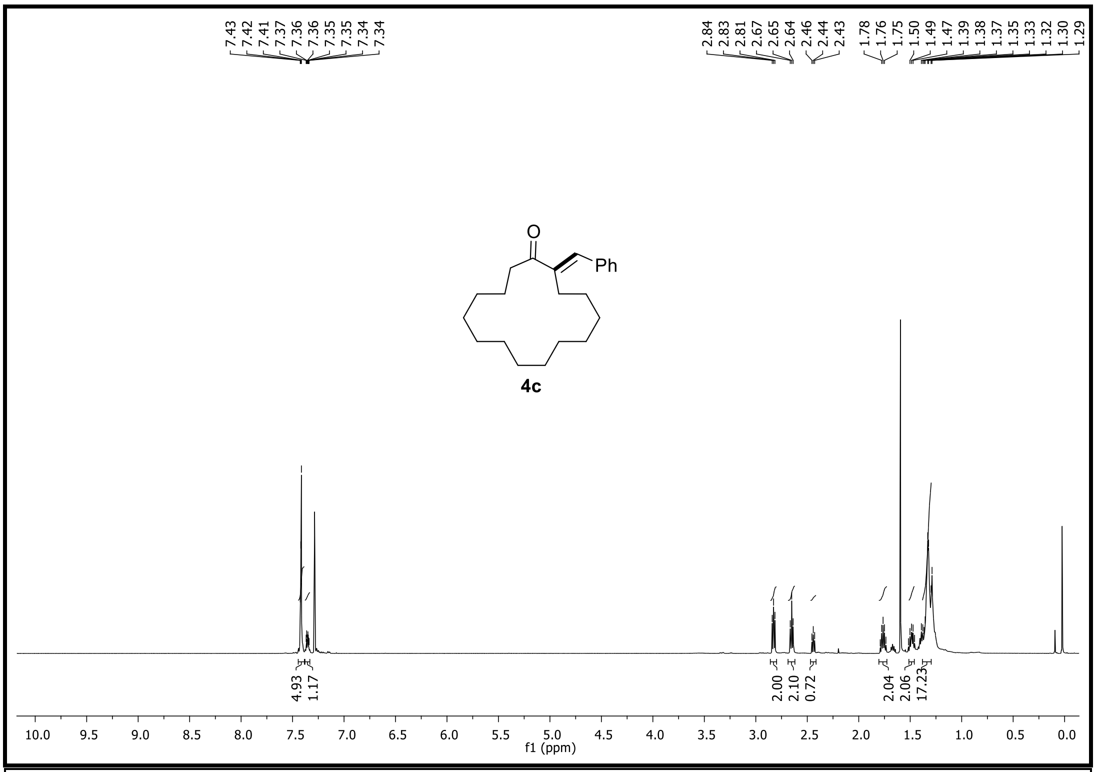
7.43
7.42
7.41
7.37
7.36
7.36
7.35
7.34
7.34
2.84
2.83
2.81
2.67
2.65
2.64
2.46
2.44
2.43
1.78
1.76
1.75
1.50
1.49
1.47
1.39
1.38
1.37
1.35
1.33
1.32
1.30
1.29
O
Ph
4c
4.93
1.17
2.00
2.10
0.72
2.04
2.06
17.23
10.0 9.5 9.0 8.5 8.0 7.5 7.0 6.5 6.0 5.5 5.0 4.5 4.0 3.5 3.0 2.5 2.0 1.5 1.0 0.5 0.0
f1 (ppm)

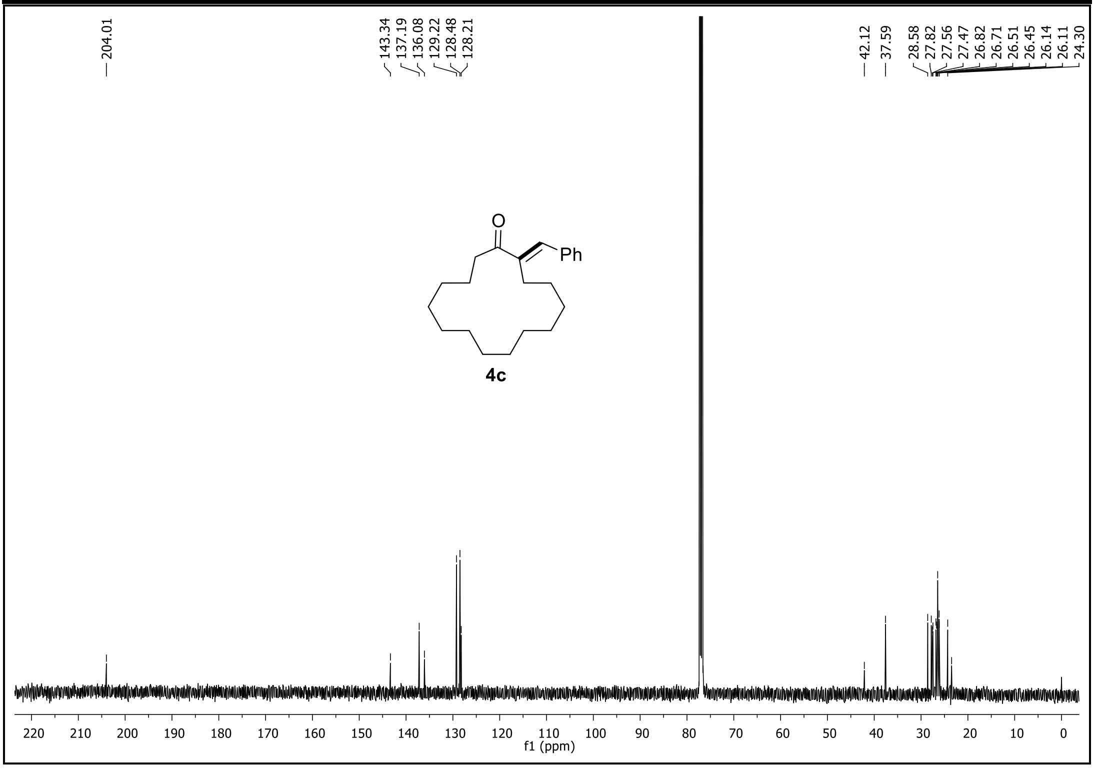
204.01
143.34
137.19
136.08
129.22
128.48
128.21
42.12
37.59
28.58
27.82
27.56
27.47
26.82
26.71
26.51
26.45
26.14
26.11
24.30
O
Ph
4c
220 210 200 190 180 170 160 150 140 130 120 110 100 90 80 70 60 50 40 30 20 10 0
f1 (ppm)

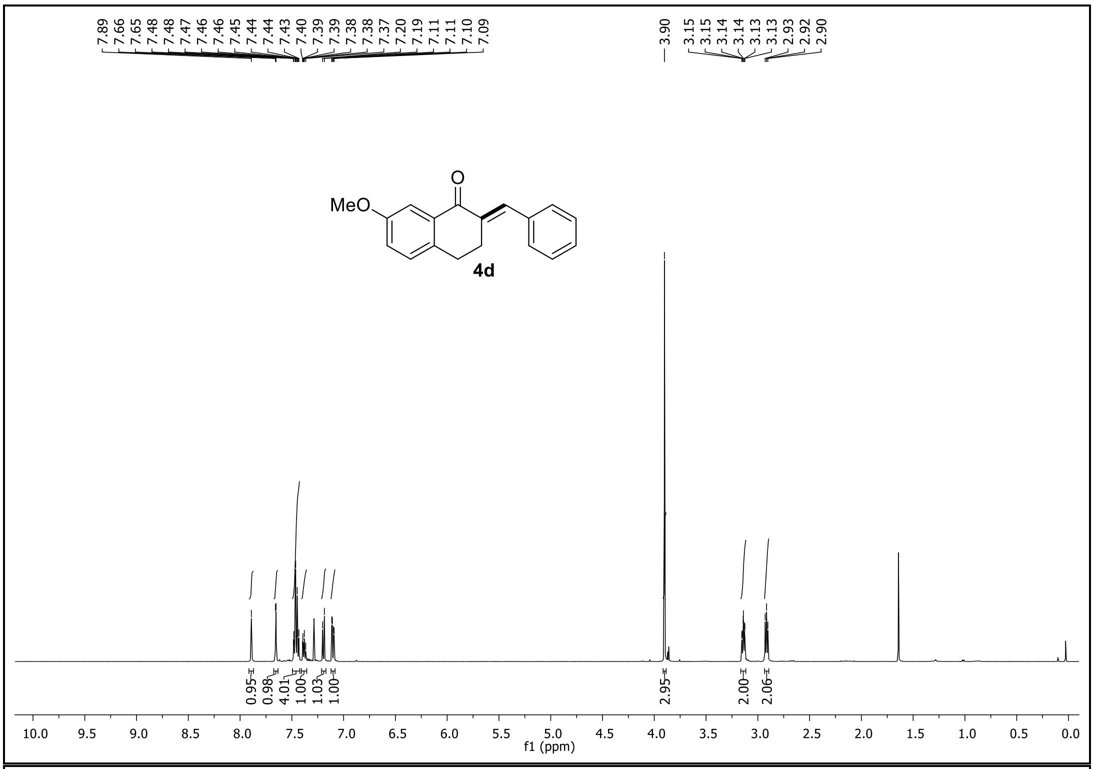
MeO
O
4d
f1 (ppm)

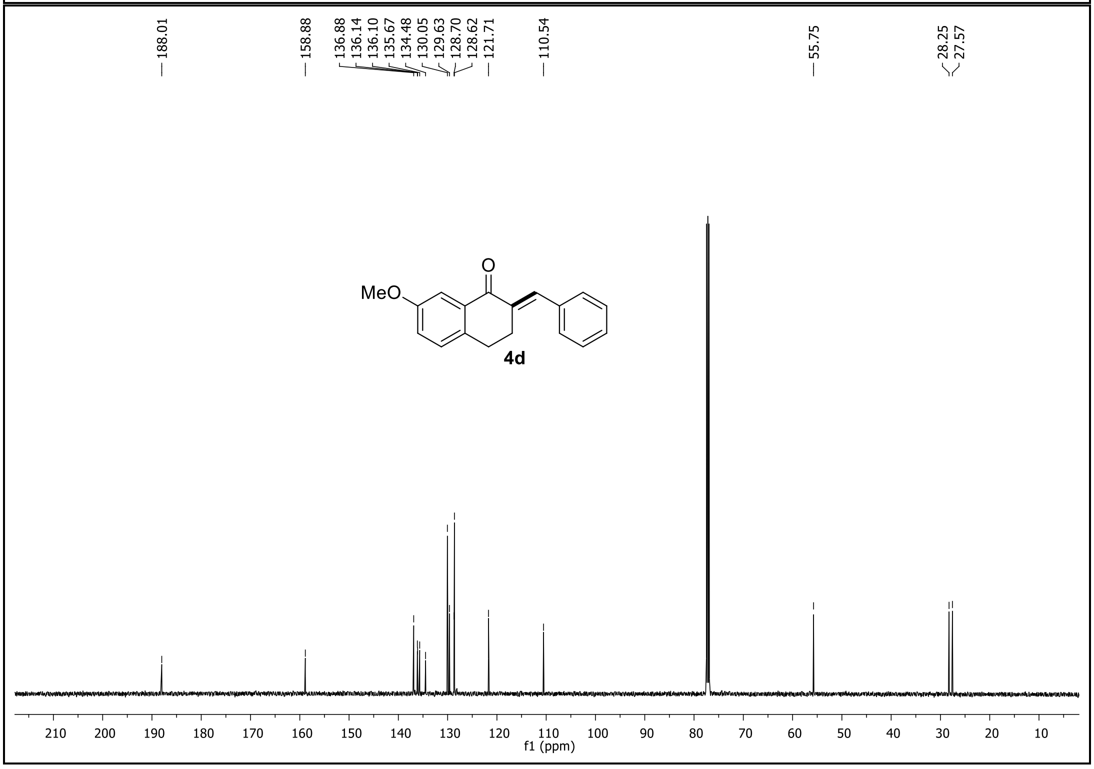
MeO
O
4d
f1 (ppm)

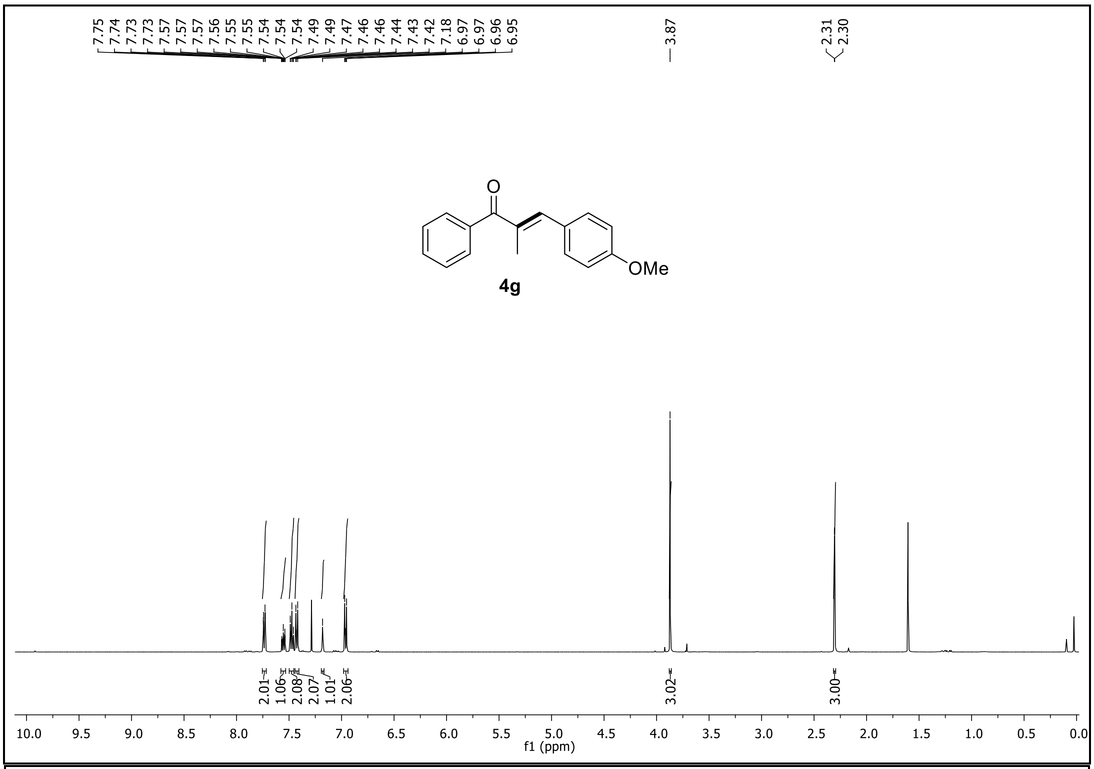
7.75
7.74
7.73
7.73
7.57
7.57
7.57
7.56
7.55
7.54
7.54
7.54
7.49
7.49
7.47
7.46
7.44
7.43
7.42
7.18
6.97
6.97
6.96
6.95
3.87
2.31
2.30
O
OMe
4g
2.01
1.06
2.08
2.07
1.01
2.06
3.02
3.00
10.0 9.5 9.0 8.5 8.0 7.5 7.0 6.5 6.0 5.5 5.0 4.5 4.0 3.5 3.0 2.5 2.0 1.5 1.0 0.5 0.0
f1 (ppm)

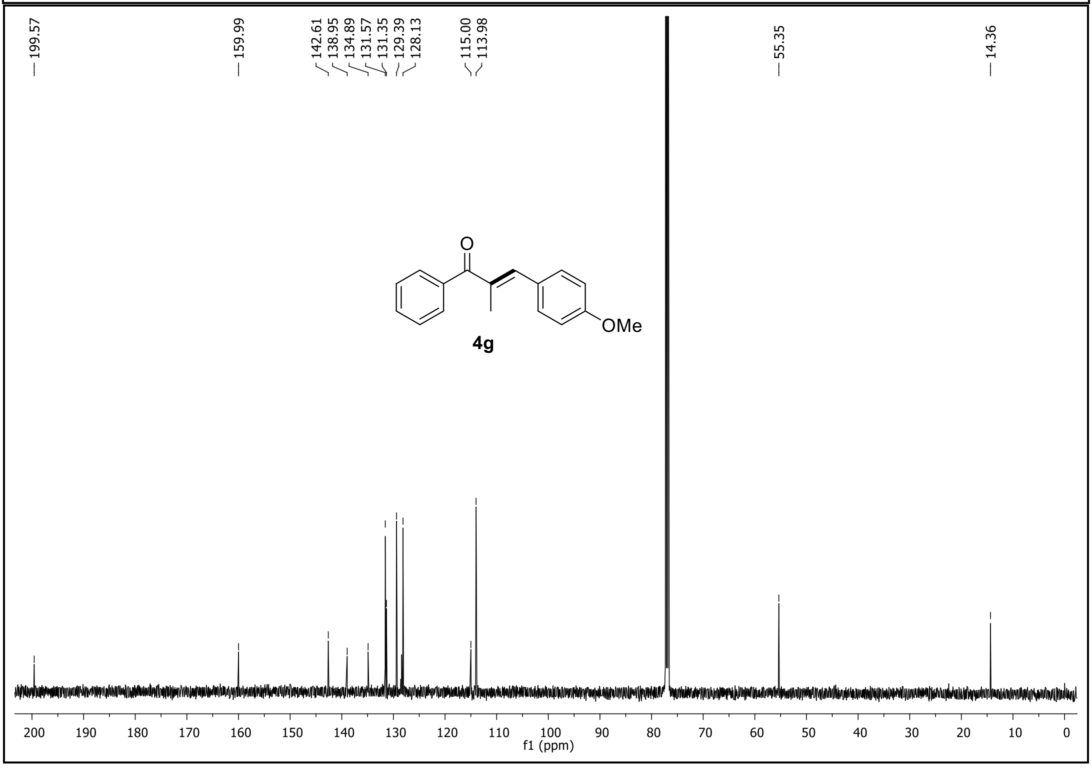
199.57
159.99
142.61
138.95
134.89
131.57
131.35
129.39
128.13
115.00
113.98
55.35
14.36
O
OMe
4g
200 190 180 170 160 150 140 130 120 110 100 90 80 70 60 50 40 30 20 10 0
f1 (ppm)

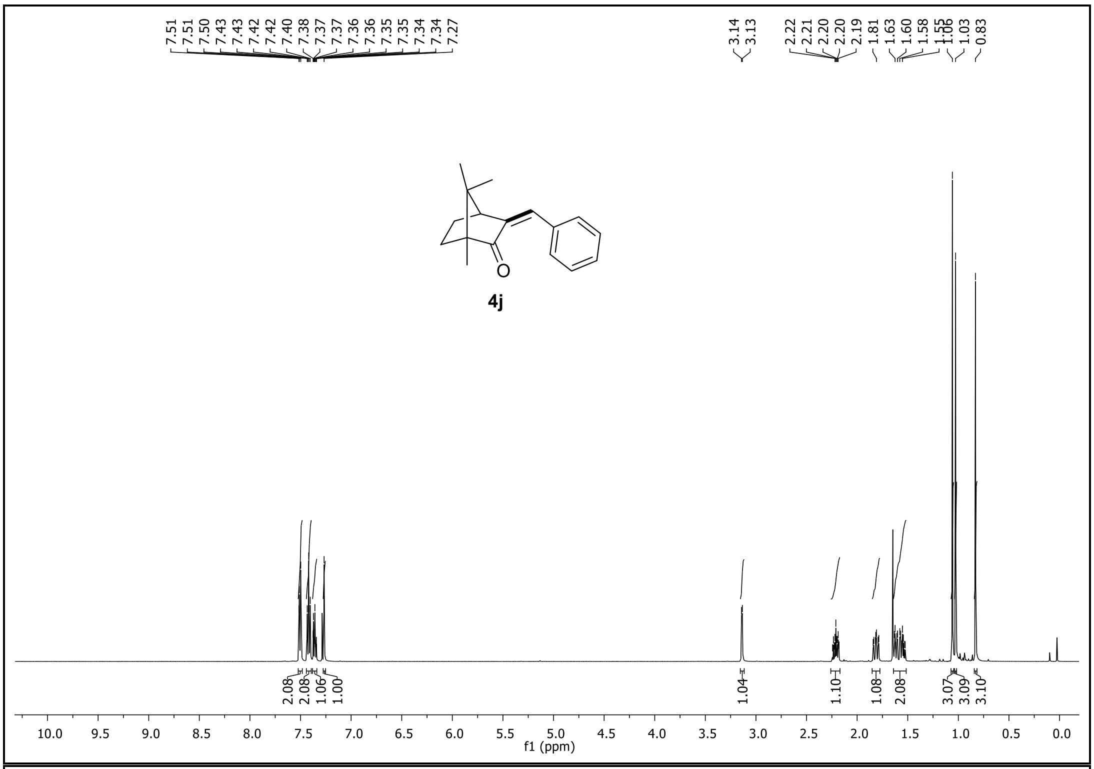
7.51
7.51
7.50
7.43
7.43
7.42
7.42
7.40
7.38
7.37
7.37
7.36
7.36
7.35
7.35
7.34
7.34
7.27
3.14
3.13
2.22
2.21
2.20
2.20
2.19
1.81
1.63
1.60
1.58
1.55
1.06
1.03
0.83
4j
2.08
2.08
1.06
1.00
1.04
1.10
1.08
2.08
3.07
3.09
3.10
10.0
9.5
9.0
8.5
8.0
7.5
7.0
6.5
6.0
5.5
5.0
4.5
4.0
3.5
3.0
2.5
2.0
1.5
1.0
0.5
0.0
f1 (ppm)

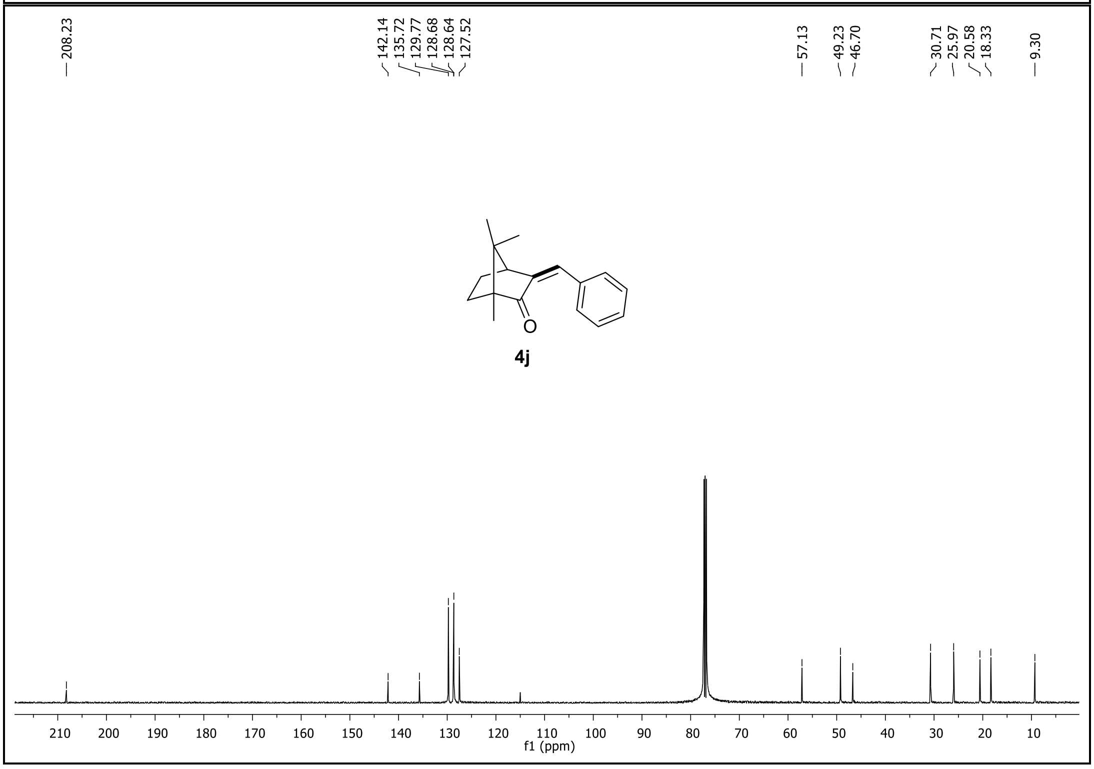
208.23
142.14
135.72
129.77
128.68
128.64
127.52
57.13
49.23
46.70
30.71
25.97
20.58
18.33
9.30
4j
210
200
190
180
170
160
150
140
130
120
110
100
90
80
70
60
50
40
30
20
10
f1 (ppm)

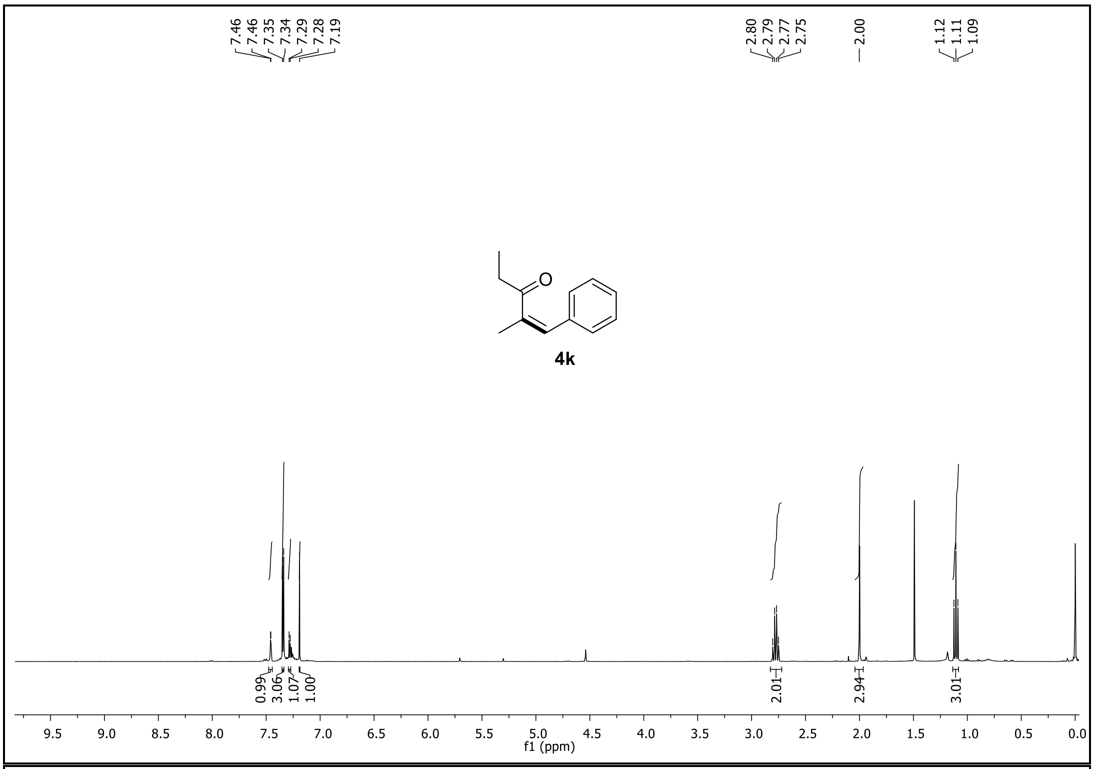
7.46
7.46
7.35
7.34
7.29
7.28
7.19
2.80
2.79
2.77
2.75
2.00
1.12
1.11
1.09
4k
0.99
3.06
1.07
1.00
2.01
2.94
3.01
9.5 9.0 8.5 8.0 7.5 7.0 6.5 6.0 5.5 5.0 4.5 4.0 3.5 3.0 2.5 2.0 1.5 1.0 0.5 0.0
f1 (ppm)

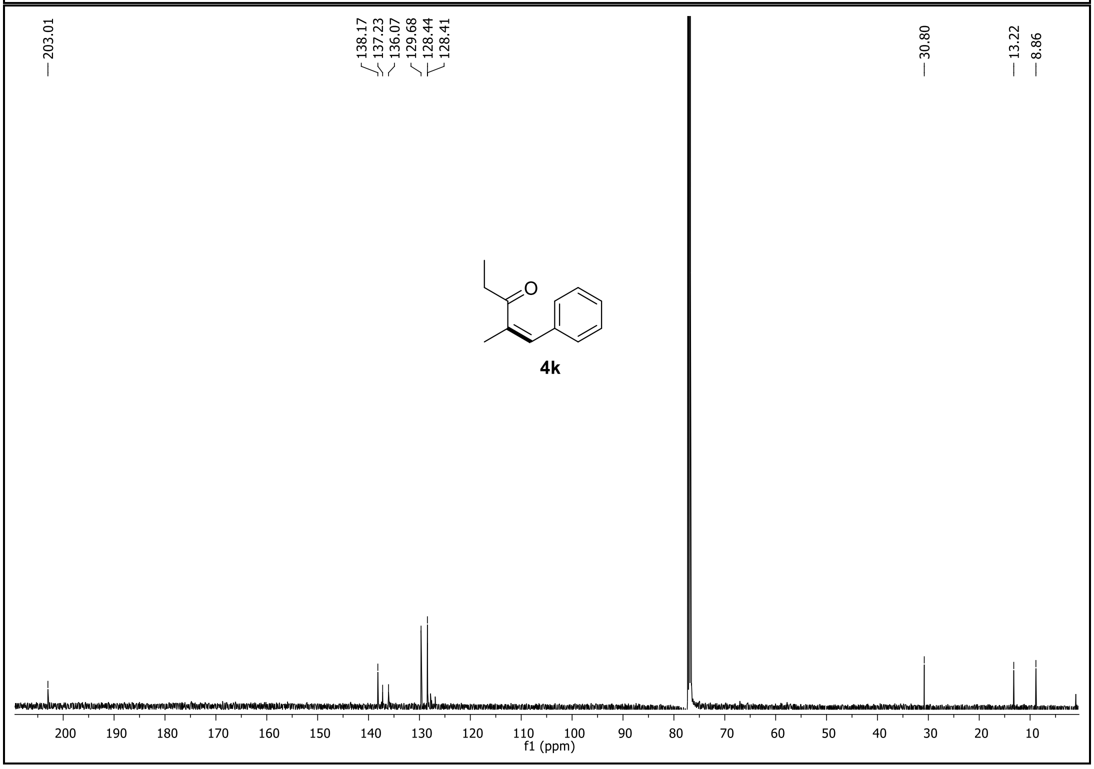
203.01
138.17
137.23
136.07
129.68
128.44
128.41
30.80
13.22
8.86
4k
200 190 180 170 160 150 140 130 120 110 100 90 80 70 60 50 40 30 20 10
f1 (ppm)
